# Gradient and GENERIC Systems in the Space of Fluxes, Applied to Reacting Particle Systems

**DOI:** 10.3390/e20080596

**Published:** 2018-08-09

**Authors:** D. R. Michiel Renger

**Affiliations:** Weierstrass Institute (WIAS), Mohrenstrasse 39, 10117 Berlin, Germany; renger@wias-berlin.de; Tel.: +49-30-20372-470

**Keywords:** large deviations, fluxes, macroscopic fluctuation theory, Onsager–Machlup, gradient structures, GENERIC, chemical reaction networks, 05.70.Ln, 82.40.Bj, 82.60.-s, 2.20.Db, 82.20.Fd, 60F10, 60J27, 80A30, 82C22, 82C35

## Abstract

In a previous work we devised a framework to derive generalised gradient systems for an evolution equation from the large deviations of an underlying microscopic system, in the spirit of the Onsager–Machlup relations. Of particular interest is the case where the microscopic system consists of random particles, and the macroscopic quantity is the empirical measure or concentration. In this work we take the particle flux as the macroscopic quantity, which is related to the concentration via a continuity equation. By a similar argument the large deviations can induce a generalised gradient or GENERIC system in the space of fluxes. In a general setting we study how flux gradient or GENERIC systems are related to gradient systems of concentrations. This shows that many gradient or GENERIC systems arise from an underlying gradient or GENERIC system where fluxes rather than densities are being driven by (free) energies. The arguments are explained by the example of reacting particle systems, which is later expanded to include spatial diffusion as well.

## 1. Introduction

By the Boltzmann–Einstein relation, the free energy of a system is inherently related to the fluctuations of an underlying microscopic particle system. In this sense, two systems with the same macroscopic behaviour can be driven by completely different free energies, if their corresponding microscopic systems are different. Therefore, one of the main objectives of (equilibrium) statistical mechanics is to derive the “physically correct” macroscopic free energy from fluctuations in microscopic systems. A similar principle can be applied to systems that evolve over time, where dynamic fluctuations may lead to a gradient flow, driven by the free energy. For stochastically reversible systems and close to equilibrium this is the classic Onsager–Machlup theory [1,2]. Such relations are known to hold for many reversible dynamics, not necessarily close to equilibrium [3]. More recently, it was shown that microscopic reversibility always implies the emergence of a macroscopic gradient flow [4], but in this generality one needs to allow for so-called generalised gradient flows. In brief, a generalised gradient structure (GGS) is defined by a possibly non-linear relation between velocities and affinities. Although there exist non-reversible models that lead to macroscopic gradient flows [5], these are considered non-typical; so in order to understand systems with non-reversible microscopic fluctuations, one needs to look for even further (thermodynamically consistent) generalisations of a gradient flow.

One such generalisation is a class of equations called GENERIC [6]. These equations can be seen as a coupling between a gradient flow of some non-increasing free energy (or non-decreasing entropy) and a Hamiltonian system of some conserved energy. One assumes that the two structures are in a sense orthogonal to each other, which then guarantees that also for the coupled evolution, the free energy is non-decreasing and the Hamiltonian energy is conserved. We explain this concept in more details in Section 3.3 and Section 4.2.

The first GENERIC structure that was derived from dynamical large deviations can be found in [7]. In order to pursue a similar procedure in a general setting, one again needs to allow for non-linear relations, just like generalised gradient flows. One is thus lead to study generalised GENERIC structures (GGEN) [8]. In a recent work, necessary and sufficient conditions were found under which microscopic fluctuations induce such a GGEN structure [9,10].

Naturally, many systems do not satisfy those conditions nor do they have a GGEN structure. Therefore there is a need for even more general structures that can still be given a meaningful thermodynamic interpretation. At this point we place ourselves in the context of Macroscopic Fluctuation Theory (MFT) [11]. Central to MFT is the idea that more thermodynamic properties of non-equilibrium fluctuations can be derived if, in addition to macroscopic state variables, the corresponding fluxes are taken into account. In general, fluxes hold more information than state variables due to the possible occurrence of “divergence-free” fluxes that do not alter the states.

One way in which this extra information can be exploited is to extract a generalisation of a GGS, where the affinity/driving force may no longer be the gradient of some free energy [12,13,14,15].

Another way to exploit the flux fluctuations, which is pursued in this paper, is to extract GGS/GGEN structures in a larger “flux space”. The heuristics behind this is that a GGS/GGEN, even in flux space, can be interpreted as a free energy balance. If there would be work done that results in a divergence-free flux, then one might expect an gap in the energy balance, so that such systems can not induce a GGS or GGEN. Could it be possible for such a system to have a GGS/GGEN structure in the flux space, when it fails to have a GGS/GGEN structure in the state space? The main point of this paper is that this is generally impossible. We will see in Theorem 3 that if the fluctuations induce a GGS or GGEN in the space of fluxes, this is, up to physical conditions, equivalent to the fluctuations inducing a GGS or GGEN in the state space.

Our leading example and main application will be that of a (non-spatial) isothermal chemical reaction network, as studied in [16]. In Section 2 we recall the main arguments from that paper, applied to concentrations undergoing reactions. In Section 3 we expand these ideas and show how they can be applied to reaction fluxes. It turns out that reactions that occur on a faster time scale may give rise to a GGEN Hamiltonian part. Based on this example, we then develop an abstract theory about induced GGSs and GGENs in flux space, in Section 4. To show the generality of these principles, we then show in Section 5 how the theory applies to transport fluxes in a diffusing particles model, and in Section 6 we combine the arguments from Section 3 and Section 6 to derive results for transport and reaction fluxes in a simple reaction-diffusion model.

## 2. Leading Example 1: Chemical Reactions

In this section we explain the main concepts, mostly by reiterating the arguments of [4] and [16]. In particular, we argue that large deviations/fluctuations provide a “physically correct” GGS for the evolution of concentrations undergoing chemical reactions.

Consider a network of isothermal chemical reactions, for example:

$$\begin{array}{ccc} 2\mathrm{Na}+{\mathrm{Cl}}_{2}\to 2\mathrm{NaCl} & \mathrm{and} & 2\mathrm{NaCl}\to 2\mathrm{Na}+{\mathrm{Cl}}_{2}. \end{array}$$

We denote the set of species by Y (in this example $\left\{\mathrm{Na},{\mathrm{Cl}}_{2},\mathrm{NaCl}\right\}$) and the set of reactions by R; for each reaction *r* we consider a forward and backward reaction (so that here R consists of one element). The stoichiometric coefficients are denoted by ${\left(\alpha_{r,y}\right)}_{y\in Y}$ for the forward reactants (here (2,1,0)) and ${\left(\beta_{r,y}\right)}_{y\in Y}$ for the forward products (here (0,0,2)), which yields the state change matrix $\Gamma :=\left(\gamma_{r,y}\right):=\left(\beta_{r,y}-\alpha_{r,y}\right)$. The evolution of the concentrations $\rho_{t}\in R^{Y}$ is then described by the *Reaction Rate Equation*,

(1) $${\dot{\rho }}_{t}=\sum_{r\in R}\gamma_{r}\left(k_{r,\mathrm{fw}}\left(\rho_{t}\right)-k_{r,\mathrm{bw}}\left(\rho_{t}\right)\right)=\Gamma \bar{k}\left(\rho_{t}\right),$$

with concentration-dependent reaction rates ${\bar{k}}_{r}:=k_{r,\mathrm{fw}}-k_{r,\mathrm{bw}}$.

Typically (and often in this paper), the reaction rates will be of the form

(2) $$\begin{array}{ccc} k_{r,\mathrm{fw}}\left(\rho \right):=\kappa_{r,\mathrm{fw}}\rho^{\alpha_{r}} & \mathrm{and} & k_{r,\mathrm{bw}}\left(\rho \right):=\kappa_{r,\mathrm{bw}}\rho^{\beta_{r}} \end{array}$$

for some constants $\kappa_{r,\mathrm{fw}},\kappa_{r,\mathrm{bw}}$, using the notation $\rho^{\alpha_{r}}=\prod_{y\in Y}\rho_{y}^{\alpha_{r,y}}$. In that case the network is said to be of *mass-action kinetics*. For more background on chemical reaction networks we refer to the survey [17]. More details about the fluctuations and induced GGSs for chemical reaction networks can be found in [16]; for completeness we shall recall these results in this section.

To notationally stress the similarity and differences between different concepts throughout this paper, we shall always denote net quantities by a bar (y¯), and we distinguish functionals on state space from functionals on flux space by a hat (y^). In this section we study concentrations only, which we consider to be states.

### 2.1. Reacting Particle System

A classical microscopic particle system underlying the evolution (1) is the following [18]. Let *V* be a large, well-mixed volume that contains, at time *t*, a total number $N_{t}^{\left(V\right)}$ of particles of species $Y_{t,i}^{\left(V\right)}$, $i=1,\cdots ,N_{t}^{\left(V\right)}$. A reaction *r* occurs randomly with some propensities (jump rates) $\lambda_{r,\mathrm{fw}}^{\left(V\right)}\left(\rho \right),\lambda_{r,\mathrm{bw}}^{\left(V\right)}\left(\rho \right)$. Whenever a forward reaction *r* occurs, $\alpha_{r}$ particles are removed and $\beta_{r}$ particles are created, and vice versa for a backward reaction. Hence each reaction requires a cumbersome relabelling of particles $Y_{t,i}$. It is therefore more practical to work directly with the (particles per volume) concentration $\rho_{t,y}^{\left(V\right)}:=\frac{1}{V}\#\left\{Y_{t,i}=y,\;i=1,\cdots ,N_{t}^{\left(V\right)}\right\}$. This quantity will also play the role of the macroscopic state variable. Whenever a forward or backward reaction *r* takes place, the concentration can now be simply updated by a jump $\rho_{t}^{\left(V\right)}=\rho_{t^{-}}^{\left(V\right)}\pm \frac{1}{V}\gamma_{r}$. Then the Y-dimensional vector $\rho_{t}^{\left(V\right)}$ is a Markov jump process, which satisfies the master equation

$$\begin{array}{cc} {\dot{\hat{P}}}_{t}^{\left(V\right)}\left(\rho \right)=\sum_{r\in R} & \lambda_{r,\mathrm{fw}}^{\left(V\right)}\left(\rho -\frac{1}{V}\gamma_{r}\right){\hat{P}}_{t}^{\left(V\right)}\left(\rho -\frac{1}{V}\gamma_{r}\right)-\lambda_{r,\mathrm{fw}}^{\left(V\right)}\left(\rho \right){\hat{P}}_{t}^{\left(V\right)}\left(\rho \right)+\\ & \lambda_{r,\mathrm{bw}}^{\left(V\right)}\left(\rho +\frac{1}{V}\gamma_{r}\right){\hat{P}}_{t}^{\left(V\right)}\left(\rho +\frac{1}{V}\gamma_{r}\right)-\lambda_{r,\mathrm{bw}}^{\left(V\right)}\left(\rho \right){\hat{P}}_{t}^{\left(V\right)}\left(\rho \right). \end{array}$$

It will be beneficial to work with the corresponding generator, which is the adjoint of the right-hand side of the master equation, with respect to the dual pairing $\langle {\hat{P}}_{t}^{\left(V\right)},f\rangle =\sum_{\rho }f\left(\rho \right)\;{\hat{P}}_{t}^{\left(V\right)}\left(\rho \right)$ with an arbitrary test function:

(3) $$\left({\hat{Q}}^{\left(V\right)}f\right)\left(\rho \right):=\sum_{r\in R}\lambda_{r,\mathrm{fw}}^{\left(V\right)}\left(\rho \right)\left(f\left(\rho +\frac{1}{V}\gamma_{r}\right)-f\left(\rho \right)\right)+\lambda_{r,\mathrm{bw}}^{\left(V\right)}\left(\rho \right)\left(f\left(\rho -\frac{1}{V}\gamma_{r}\right)-f\left(\rho \right)\right).$$

The propensities that are usually used in the so-called chemical master Equation [17] are derived from combinatoric considerations, and yield the mass-action kinetics in the limit [16,18]:

(4) $$\begin{array}{c} \frac{1}{V}\lambda_{r,\mathrm{fw}}^{\left(V\right)}\left(\rho \right):=\frac{1}{V}\cdot \frac{\kappa_{r,\mathrm{fw}}}{V^{\alpha_{r,\mathrm{tot}}-1}}\frac{(\rho V)!}{(\rho V-\alpha_{r})!}\overset{V\to \infty }{\to }\kappa_{r,\mathrm{fw}}\rho^{\alpha_{r}}=k_{r,\mathrm{fw}}\left(\rho \right),\\ \frac{1}{V}\lambda_{r,\mathrm{bw}}^{\left(V\right)}\left(\rho \right):=\frac{1}{V}\cdot \frac{\kappa_{r,\mathrm{bw}}}{V^{\beta_{r,\mathrm{tot}}-1}}\frac{(\rho V)!}{(\rho V-\beta_{r})!}\overset{V\to \infty }{\to }\kappa_{r,\mathrm{bw}}\rho^{\beta_{r}}=k_{r,\mathrm{bw}}\left(\rho \right), \end{array}$$

using the notation $\alpha_{r,\mathrm{tot}}:=\sum_{y\in Y}\alpha_{r,y}$ and $\alpha_{r}!:=\prod_{y\in Y}\alpha_{r,y}!$.

### 2.2. Equilibrium: Limit, Large Deviations and Free Energy

We will from now on (throughout this section) assume that the reaction network is of mass-action kinetics (2), and chemically detailed balanced, i.e., there exists a $\rho^{*}\in R_{+}^{Y}$ for which

(5) $$\kappa_{r,\mathrm{fw}}{\rho^{*}}^{\alpha_{r}}=\kappa_{r,\mathrm{bw}}{\rho^{*}}^{\beta_{r}}\;\mathrm{for}\;\mathrm{all}\;r\in R.$$

Naturally, $\rho^{*}$ is an equilibrium under the deterministic evolution (1). It should be stressed that, given an initial concentration $\rho_{0}$, both the deterministic evolution and the stochastic model is confined to the “stoichiometric compatibility class” $\rho_{0}+\mathrm{Ran}\Gamma =\left\{\rho_{0}+\Gamma w:w\in R_{+}^{R}\right\}$. Therefore, it is not clear whether this equilibrium $\rho^{*}$ lies within the compatibility class that corresponds to the initial concentration. However, if there exists a detailed balanced concentration $\rho^{*}$, then there exists a unique detailed balanced concentration within each such class, see [17] and the references therein. Without loss of generality, we can therefore implicitly assume that the detailed balanced equilibrium is unique, and lies within the correct compatibility class.

Under Assumption (5), the invariant distribution of the stochastic model is known to be [17]:

$${\hat{P}}_{\infty }^{\left(V\right)}\left(\rho \right)=\prod_{y\in Y}\frac{{\left(V\rho_{y}^{*}\right)}^{V\rho_{y}}}{(V\rho_{y})!}e^{-V\rho_{y}^{*}}.$$

Letting V→∞, this invariant distribution concentrates on the equilibrium state:

$${\hat{P}}_{\infty }^{\left(\infty \right)}\left(\rho \right)=\left\{\begin{array}{cc} 1, & \rho =\rho^{*},\\ 0, & \mathrm{otherwise}. \end{array}\right.$$

One can then extract the free energy by considering the corresponding large deviations, i.e., the exponential rate which with ${\hat{P}}_{\infty }^{\left(V\right)}$ converges to zero. Indeed, by Stirling’s formula,

(6) $$-\frac{1}{V}\log{\hat{P}}_{\infty }^{\left(V\right)}\left(\rho \right)\overset{V\to \infty }{\to }\sum_{y\in Y}\rho_{y}\log\rho_{y}\rho_{y}^{*}-\rho_{y}+\rho_{y}^{*}=:h\left(\rho |\rho^{*}\right).$$

Such limit is known as a large-deviation principle; the function on the right characterises the stochastic cost of microscopic fluctuations. If the reaction rates are related to an internal energy via Arrhenius’ law, then the expression $h(\rho |\rho^{*})$ is really the Helmholtz free energy, apart from a normalisation term and a constant scaling, as explained in more detail in (Section 2.2 & 2.3, [16]).

### 2.3. Dynamics: Limit and Large Deviations

Observe that in the microscopic model, the process speeds up as *V* increases with order *V* while the jump sizes are of size 1/V. Therefore by (4), as V→∞ the generator ${\hat{Q}}^{\left(V\right)}$ converges to the limit generator,

$$\left({\hat{Q}}^{\left(\infty \right)}f\right)\left(\rho \right):=\sum_{r\in R}{\bar{k}}_{r}\left(\rho \right)\nabla f\left(\rho \right)\cdot \gamma_{r}.$$

Since this generator depends on the test function *f* through ∇f(ρ) only, we can make the ansatz that the limit process is deterministic ${\hat{P}}_{t}^{\left(\infty \right)}\left(\tilde{\rho }\right)=\delta_{\rho_{t}}\left(\tilde{\rho }\right)$, for some curve $\rho_{\left(\cdot \right)}$. Plugging this into the definition of the generator yields:

$$\nabla f\left(\rho_{t}\right)\cdot {\dot{\rho }}_{t}=\partial_{t}f\left(\rho_{t}\right)=\partial_{t}\langle {\hat{P}}_{t}^{\left(\infty \right)},f\rangle =\langle {\hat{P}}_{t}^{\left(\infty \right)},Q^{\left(\infty \right)}f\rangle =\left({\hat{Q}}^{\left(\infty \right)}f\right)\left(\rho_{t}\right)=\nabla f\left(\rho_{t}\right)\cdot \sum_{r\in R}{\bar{k}}_{r}\left(\rho_{t}\right)\gamma_{r}.$$

As this relation holds for any test function *f*, we see that the ansatz was justified if the postulated curve $\rho_{t}$ satisfies the Reaction Rate Equation (1). Hence the stochastic process $\rho_{t}^{\left(V\right)}$ converges (pathwise in probability) to the deterministic solution $\rho_{t}$ of the Reaction Rate Equation.

Similar to the calculation of the fluctuations of the equilibrium (6), we can study the large deviations of the path probabilities ${\hat{P}}^{\left(V\right)}$; this is known as a dynamic large-deviation principle. These dynamical fluctuations can be formally calculated with the framework of [19]. To this aim we study the non-linear generator:

$$\begin{array}{cc} \left({\hat{ℋ}}^{\left(V\right)}f\right)\left(\rho \right) & :=\frac{1}{V}e^{-Vf(\rho )}\left({\hat{Q}}^{\left(V\right)}e^{Vf}\right)\left(\rho \right)\\ & =\sum_{r\in R}\frac{1}{V}\lambda_{r,\mathrm{fw}}^{\left(V\right)}\left(\rho \right)\left(e^{Vf\left(\rho +\frac{1}{V}\gamma_{r}\right)-Vf\left(\rho \right)}-1\right)+\frac{1}{V}\lambda_{r,\mathrm{bw}}^{\left(V\right)}\left(\rho \right)\left(e^{Vf\left(\rho -\frac{1}{V}\gamma_{r}\right)-Vf\left(\rho \right)}-1\right)\\ & \overset{V\to \infty }{\to }\sum_{r\in R}k_{r,\mathrm{fw}}\left(\rho \right)\left(e^{\nabla f\left(\rho \right)\cdot \gamma_{r}}-1\right)+k_{r,\mathrm{bw}}\left(\rho \right)\left(e^{-\nabla f\left(\rho \right)\cdot \gamma_{r}}-1\right). \end{array}$$

As before, the limit depends on the test function through ∇f(ρ) only, which is consistent with the fact that the limit is deterministic. We then define, by a slight abuse of notation,

(7) $$\begin{array}{ccc} \hat{ℋ}\left(\rho ,\xi \right) & :=\sum_{r\in R}k_{r,\mathrm{fw}}\left(\rho \right)\left(e^{\xi \cdot \gamma_{r}}-1\right)+k_{r,\mathrm{bw}}\left(\rho \right)\left(e^{-\xi \cdot \gamma_{r}}-1\right), & \mathrm{and} \end{array}$$

(8) $$\begin{array}{cc} \hat{ℒ}\left(\rho ,s\right) & :=\underset{\xi \in R^{Y}}{\sup}\xi \cdot s-\hat{ℋ}\left(\rho ,\xi \right). \end{array}$$

The dynamic large-deviation principle now states that

(9) $${\mathrm{Prob}}^{\left(V\right)}\;\left(\rho_{\left(\cdot \right)}^{\left(V\right)}\approx \rho_{\left(\cdot \right)}\right)\overset{V\to \infty }{∼}e^{-V\int_{0}^{T}\;\hat{ℒ}\left(\rho_{t},{\dot{\rho }}_{t}\right)\;dt}.$$

The rigorous definition of the large-deviation principle, the heuristics behind this method, and the rigorous proof of this statement is all beyond the scope of this paper. For the precise details we refer to [19], and for the rigorous proof for this particular system (by more classical methods) to [20,21,22]. For the sake of brevity, we assume that the randomness in the initial condition is sufficiently small (e.g., deterministic) so that we do not obtain initial fluctuations.

**Remark** **1.** *The function *(8)* is implicitly defined as a supremum; although the supremum can be calculated explicitly, this leads to very cumbersome expressions. However, it does have a dual formulation in terms of a minimisation problem:*

$$\hat{ℒ}\left(\rho ,s\right)=\underset{\begin{array}{c} j_{\mathrm{fw}},j_{\mathrm{bw}}\in R_{+}^{R}:\\ \Gamma (j_{\mathrm{fw}}-j_{\mathrm{bw}})=s \end{array}}{\inf}\;h\left(j_{\mathrm{fw}}|k_{\mathrm{fw}}\left(\rho \right)\right)+h\left(j_{\mathrm{bw}}|k_{\mathrm{bw}}\left(\rho \right)\right),$$

*where $h\left(j|k\right):=\sum_{r\in R}j_{r}\log\left(j_{r}/k_{r}\right)-j_{r}+k_{r}$, similar to *(6)*. Although the relative entropy h appears in both expressions, they should not be confused: $h(\rho |\rho^{*})$ is an equilibrium rate whereas h(j|k(ρ)) is a dynamic quantity. We shall see later on that the latter can be directly (without the infimum) be interpreted as a large-deviation rate, where the variable j is a reaction flux.*

### 2.4. GGS, Energy Balance, and Relation with Fluctuations

The (naive) aim is to rewrite the macroscopic equation as a gradient flow of some free energy F:

(10) $${\dot{\rho }}_{t}=-\hat{K}\left(\rho_{t}\right)\nabla \hat{F}\left(\rho_{t}\right)=:-{\mathrm{grad}}_{\rho_{t}}\hat{F}\left(\rho_{t}\right),$$

where $\hat{K}\left(\rho \right)$ is some linear symmetric, positive definite (linear response) operator that maps thermodynamic forces to velocities. Mathematically, this operator can be interpreted as the inverse of the metric tensor of some manifold, so that the right-hand side is the gradient on this manifold.

Clearly, (10) is equivalent to requiring

$$0=\frac{1}{2}{∥{\dot{\rho }}_{t}+\hat{K}\left(\rho_{t}\right)\nabla \hat{F}\left(\rho_{t}\right)∥}_{\hat{K}{\left(\rho_{t}\right)}^{-1}}^{2}=\frac{1}{2}{∥{\dot{\rho }}_{t}∥}_{\hat{K}{\left(\rho_{t}\right)}^{-1}}^{2}+\frac{1}{2}{∥\nabla \hat{F}\left(\rho_{t}\right)∥}_{\hat{K}\left(\rho_{t}\right)}^{2}+\nabla \hat{F}\left(\rho_{t}\right)\cdot {\dot{\rho }}_{t},$$

if we set ${∥\xi ∥}_{\hat{K}\left(\rho \right)}^{2}:=\langle \xi ,\hat{K}\left(\rho \right)\xi \rangle$ and ${∥s∥}_{\hat{K}{\left(\rho \right)}^{-1}}^{2}:=\langle s,\hat{K}{\left(\rho \right)}^{-1}s\rangle$. Integrated over a time interval (0,T), this reads

$$0=\int_{0}^{T}\;\left(\frac{1}{2}{∥{\dot{\rho }}_{t}∥}_{\hat{K}{\left(\rho_{t}\right)}^{-1}}^{2}+\frac{1}{2}{∥\nabla \hat{F}\left(\rho_{t}\right)∥}_{\hat{K}\left(\rho_{t}\right)}^{2}\right)\;dt+\hat{F}\left(\rho_{T}\right)-\hat{F}\left(\rho_{0}\right).$$

The last two terms describe the free energy loss (or entropy production), and the first two terms describe the dissipation; as such this equation represents a free energy balance. (For a linear gradient flow (10), $\frac{1}{2}{∥{\dot{\rho }}_{t}∥}_{\hat{K}{\left(\rho_{t}\right)}^{-1}}^{2}+\frac{1}{2}{∥\nabla \hat{F}\left(\rho_{t}\right)∥}_{\hat{K}\left(\rho_{t}\right)}^{2}={∥{\dot{\rho }}_{t}∥}_{\hat{K}{\left(\rho \right)}^{-1}}^{2}$; hence the dissipation can be seen as a kinetic energy. This is however no longer true for general GGSs).

Observe that this expression is always non-negative, and is 0 exactly on the gradient flow (10). Moreover, we see that this expression has the same dimension as $\hat{F}$, the free energy; it is indeed the free energy cost to deviate from the macroscopic dynamics. This interpretation shows that this cost should be equal to the cost $\int_{0}^{T}\;\hat{ℒ}\left(\rho_{t},{\dot{\rho }}_{t}\right)\;dt$ of microscopic fluctuations (9).

This is in many cases, and particularly in this case of chemical reactions, impossible. Since the large-deviation function (8) is non-quadratic, one should allow for non-quadratic dissipation terms. We therefore replace the two squared norms by a pair of dual *dissipation potentials*:

(11) $$\begin{array}{cc} 0 & =\int_{0}^{T}\;\left(\hat{\Psi }\left(\rho_{t},{\dot{\rho }}_{t}\right)+{\hat{\Psi }}^{*}\left(\rho_{t},-\nabla \hat{F}\left(\rho_{t}\right)\right)+\nabla \hat{F}\left(\rho_{t}\right)\cdot {\dot{\rho }}_{t}\right)\;dt \end{array}$$

(12) $$\begin{array}{c} \;=\int_{0}^{T}\;\left(\hat{\Psi }\left(\rho_{t},{\dot{\rho }}_{t}\right)+{\hat{\Psi }}^{*}\left(\rho_{t},-\nabla \hat{F}\left(\rho_{t}\right)\right)\right)\;dt+\hat{F}\left(\rho_{T}\right)-\hat{F}\left(\rho_{0}\right), \end{array}$$

where, as in the quadratic case, the potentials are convex duals of each other, i.e., ${\hat{\Psi }}^{*}\left(\rho ,\xi \right):=\sup_{s}\xi \cdot s-\hat{\Psi }\left(\rho ,s\right)$ and $\hat{\Psi }\left(\rho ,s\right)=\sup_{\xi }\xi \cdot s-{\hat{\Psi }}^{*}\left(\rho ,\xi \right)$. Moreover, we assume that $\hat{\Psi }$ and ${\hat{\Psi }}^{*}$ are both non-negative; from (12) we then see that the free energy $\hat{F}$ is non-increasing on the flow.

The right-hand side of (12) is always non-negative, since ${\hat{\Psi }}^{*}\left(\rho_{t},-\nabla \hat{F}\left(\rho_{t}\right)\right)\geq -\nabla \hat{F}\left(\rho_{t}\right)\cdot {\dot{\rho }}_{t}-\hat{\Psi }\left(\rho_{t},{\dot{\rho }}_{t}\right)$ by definition of the convex dual. Therefore, the right-hand side of (12) can only be 0 if $-\nabla \hat{F}\left(\rho_{t}\right)$ is minimal in the right-hand side. By differentiation we then get that (12) implies:

$${\dot{\rho }}_{t}=\nabla_{\xi }{\hat{\Psi }}^{*}\left(\rho_{t},-\nabla \hat{F}\left(\rho_{t}\right)\right).$$

We call such equation a *generalised gradient flow*, and the underlying structure $\left(R^{Y},\hat{\Psi },\hat{F}\right)$ a *generalised gradient structure (GGS)*. Note that the generalisation with respect to (10) lies in the fact that we allow for a non-linear relation between forces and velocities. We moreover say that a gradient structure is *induced* by a cost function $\hat{ℒ}$ whenever $\hat{ℒ}\left(\rho ,s\right)=\hat{\Psi }\left(\rho ,s\right)+{\hat{\Psi }}^{*}\left(\rho ,-\nabla \hat{F}\left(\rho \right)\right)+\nabla \hat{F}\left(\rho \right)\cdot s$.

In Section 4 we will recall the relation between fluctuation costs and GGSs, as described in [4]. Applied to the current setting of chemical reactions, we reiterate the following result from [16]. If we again assume mass-action kinetics (2) and chemical detailed balance (5), then there exists a unique GGS $\left(R^{Y},\hat{\Psi },\hat{F}\right)$ induced by the large-deviation cost $\hat{ℒ}$ from (8), where

(13) $$\begin{array}{ccc} \hat{F}\left(\rho \right):=\frac{1}{2}h\left(\rho |\rho^{*}\right), & \mathrm{and} & {\hat{\Psi }}^{*}\left(\rho ,\xi \right):=\sum_{r\in R}{\hat{\sigma }}_{r}\left(\rho \right)\left(\cosh(\xi \cdot \gamma_{r})-1\right), \end{array}$$

with ${\hat{\sigma }}_{r}\left(\rho \right):=2\sqrt{k_{r,\mathrm{fw}}\left(\rho \right)k_{r,\mathrm{bw}}\left(\rho \right)}$. Since ${\hat{\Psi }}^{*}$ appears as a sum, the expression for Ψ becomes a so-called inf-convolution where all reactions are strongly intertwined. For more details on these inf-convolutions and the factor 1/2 in front of the free energy, we again refer to (Section 3.4, [16]).

## 3. Leading Example 2: Fast-Slow Reaction Fluxes

As mentioned in the introduction, the motivation behind the current paper is to search for thermodynamically consistent structures for systems that are not detailed balanced. The idea is that we increase the space by taking fluxes into account. However, in order to see the connection between structures in flux space and structures in state space, we dedicate this section to an example that is “almost” detailed balanced. More general systems will be dealt with when discussing the general theory in Section 4.

### 3.1. Reacting Particle System and Macroscopic Equation

We consider a system of fast and slow chemical reactions, $R=R_{\mathrm{slow}}\cup R_{\mathrm{fast}}$, where the slow reactions are assumed to be of mass-action kinetics and detailed balanced, as in the previous section. By contrast, we will not assume anything of the like for the fast reactions, neither shall we assume that each reaction consist of a forward and a backward reaction. However, in the microscopic model, we shall assume that the fast reactions happen on a faster time-scale, i.e.,

(14) $$\frac{1}{V^{2}}\lambda_{r}^{\left(V\right)}\left(\rho \right)\to {\tilde{k}}_{r}\left(\rho \right)\;\mathrm{for}\;r\in R_{\mathrm{fast}},$$

but their effect on the concentrations is smaller, i.e., $\rho^{\left(V\right)}\left(t\right)=\rho^{\left(V\right)}\left(t^{-}\right)+\frac{1}{V^{2}}\gamma_{r}$ whenever a reaction $r\in R_{\mathrm{fast}}$ occurs at time *t*. To notationally distinguish the two time scales we use a tilde (r˜) to denote one-way fast quantities. Let us briefly mention that the additional fast reactions are not essential to see the relation between structures in flux and state space; they just lead to a richer example that is interesting in its own right.

The main idea is now to increase the state space by bookkeeping the events in the microscopic system, in this case, by counting the number of reactions that have occurred up to a given time. With the right scaling, this defines the following *integrated reaction fluxes*:

$$\begin{array}{cc} {\bar{W}}_{t,r}^{\left(V\right)}:=\frac{1}{V}\#\left\{\mathrm{forward}\;\mathrm{reactions}\;r\;\mathrm{occurred}\;\mathrm{in}\;(0,t)\right\}\\ \;-\frac{1}{V}\#\left\{\mathrm{backward}\;\mathrm{reactions}\;r\;\mathrm{occurred}\;\mathrm{in}\;(0,t)\right\}, & r\in R_{\mathrm{slow}},\\ {\tilde{W}}_{t,r}^{\left(V\right)}:=\frac{1}{V^{2}}\#\left\{\mathrm{reactions}\;r\;\mathrm{occurred}\;\mathrm{in}\;(0,t)\right\}, & r\in R_{\mathrm{fast}}, \end{array}$$

and to shorten notation we sometimes write

(15) $$W_{t}^{\left(V\right)}:=\left({\left({\bar{W}}_{t,r}^{\left(V\right)}\right)}_{r\in R_{\mathrm{slow}}},{\left({\tilde{W}}_{t,r}^{\left(V\right)}\right)}_{r\in R_{\mathrm{fast}}}\right).$$

The term “integrated” signifies that these fluxes are cumulative over time. This simplifies the microscopic analysis since the corresponding process is Markovian; on a macroscopic scale only the time-derivatives will play a role. We also mention that we consider net rather than one-way slow fluxes, else the slow dynamics would not induce any force or gradient structure, see (Section 4.6, [15]).

We shall always assume that the initial condition is known (deterministically) a priori, so that there always holds:

$$\rho_{t}^{\left(V\right)}=\phi^{\left(V\right)}\left[W_{t}^{\left(V\right)}\right]:=\rho_{0}^{\left(V\right)}+\Gamma W_{t}^{\left(V\right)}.$$

In this sense the integrated fluxes encode more information than the concentrations. The equation above relates the change in concentration to the integrated fluxes, and can thus be interpreted as a *continuity equation*.

The integrated fluxes are again a Markov process, with generator (cf. Equation (3)):

$$\begin{array}{cc} \left(Q^{\left(V\right)}f\right)\left(w\right):= & \sum_{r\in R_{\mathrm{slow}}}\lambda_{r,\mathrm{fw}}^{\left(V\right)}\left(\phi^{\left(V\right)}\left[w\right]\right)\left(f\left(w+\frac{1}{V}{𝟙}_{r}\right)-f\left(w\right)\right)+\lambda_{r,\mathrm{bw}}^{\left(V\right)}\left(\phi^{\left(V\right)}\left[w\right]\right)\left(f\left(w-\frac{1}{V}{𝟙}_{r}\right)-f\left(w\right)\right)\\ & +\sum_{r\in R_{\mathrm{fast}}}\lambda_{r}^{\left(V\right)}\left(\phi^{\left(V\right)}\left[w\right]\right)\left(f\left(w+\frac{1}{V^{2}}{𝟙}_{r}\right)-f\left(w\right)\right). \end{array}$$

### 3.2. Limit and Large Deviations

We now mimic the arguments of Section 2.3, but now in the space of fluxes. Let $\rho_{0}^{\left(V\right)}\to \rho_{0}$, and so $\phi^{\left(V\right)}\left[w\right]\to \phi \left[w\right]:=\rho_{0}+\Gamma w$. Then by the same argument as in Section 2.3, using the scalings (4) and (14), one finds that as V→∞, the random process $W_{t}^{\left(V\right)}$ converges (pathwise in probability) to the solution of the macroscopic equations:

(16) $$\begin{array}{ccc} {\dot{\bar{w}}}_{t,r}={\bar{k}}_{r}\left(\phi [w_{t}]\right),\;r\in R_{\mathrm{slow}}, & \mathrm{and} & {\dot{\tilde{w}}}_{t,r}={\tilde{k}}_{r}\left(\phi [w_{t}]\right),\;r\in R_{\mathrm{fast}}, \end{array}$$

again using the notation (15). Indeed, combining these equations leads to the macroscopic Equation ${\dot{\rho }}_{t}=\Gamma k\left(\rho_{t}\right)$ for the concentrations.

Similarly, we study the fluctuations through the non-linear generator:

(17) $$\begin{array}{cc} \left({ℋ}^{\left(V\right)}f\right)\left(w\right) & :=\frac{1}{V}e^{-Vf(w)}\left(Q^{\left(V\right)}e^{Vf}\right)\left(w\right)\\ & =\sum_{r\in R_{\mathrm{slow}}}\frac{1}{V}\lambda_{r,\mathrm{fw}}^{\left(V\right)}\left(\phi^{\left(V\right)}\left[w\right]\right)\left(e^{Vf\left(w+\frac{1}{V}{𝟙}_{r}\right)-Vf\left(w\right)}-1\right)\\ & \;+\frac{1}{V}\lambda_{r,\mathrm{bw}}^{\left(V\right)}\left(\phi^{\left(V\right)}\left[w\right]\right)\left(e^{Vf\left(w-\frac{1}{V}{𝟙}_{r}\right)-Vf\left(w\right)}-1\right)\\ & \;+\sum_{r\in R_{\mathrm{fast}}}\frac{1}{V}\lambda_{r}^{\left(V\right)}\left(\phi^{\left(V\right)}\left[w\right]\right)\left(e^{Vf\left(w+\frac{1}{V^{2}}{𝟙}_{r}\right)-Vf\left(w\right)}-1\right)\\ & \;\overset{V\to \infty }{\to }\sum_{r\in R_{\mathrm{slow}}}k_{r,\mathrm{fw}}\left(\phi [w])\right)\left(e^{\partial_{w_{r}}f\left(w\right)}-1\right)+k_{r,\mathrm{bw}}\left(\phi [w]\right)\left(e^{-\partial_{w_{r}}f\left(w\right)}-1\right)\\ & \;+\sum_{r\in R_{\mathrm{fast}}}{\tilde{k}}_{r}\left(\phi [w]\right)\partial_{w_{r}}f\left(w\right). \end{array}$$

As in Section 2.3, this limit depends on the gradient ∇f(w) only, which is consistent with the deterministic limit (16). Again we set, by a slight abuse of notation,

(18) $$\begin{array}{cc} ℋ(w,\zeta ) & :=\sum_{r\in R_{\mathrm{slow}}}k_{r,\mathrm{fw}}\left(\phi [w]\right)\left(e^{{\bar{\zeta }}_{r}}-1\right)+k_{r,\mathrm{bw}}\left(\phi [w]\right)\left(e^{-{\bar{\zeta }}_{r}}-1\right)+\sum_{r\in R_{\mathrm{fast}}}{\tilde{k}}_{r}\left(\phi [w]\right){\tilde{\zeta }}_{r}, \end{array}$$

$$\begin{array}{cc} ℒ(w,j) & :=\underset{\zeta \in R^{R}}{\sup}\zeta \cdot j-ℋ\left(w,\zeta \right)=\underset{j_{\mathrm{fw}}-j_{\mathrm{bw}}=\bar{j}}{\inf}h\left(j_{\mathrm{fw}}|k_{\mathrm{fw}}\left(\phi \left[w\right]\right)\right)+h\left(j_{\mathrm{bw}}|k_{\mathrm{bw}}\left(\phi \left[w\right]\right)\right)+\chi_{\left\{\tilde{j}=\tilde{k}\left(\phi \left[w\right]\right)\right\}}, \end{array}$$

using the notation $\chi_{\left\{\tilde{j}=\tilde{k}\left(\phi \left[w\right]\right)\right\}}=0$ if $\tilde{j}=\tilde{k}\left(\phi \left[w\right]\right)$ and *∞* otherwise. Then, the dynamic large-deviation principle for the integrated fluxes $W^{\left(V\right)}\left(t\right)$ states that (see [22] for a rigorous proof):

(19) $$\mathrm{Prob}\;\left(W_{\left(\cdot \right)}^{\left(V\right)}\approx w_{\left(\cdot \right)}\right)\overset{V\to \infty }{∼}e^{-V\int_{0}^{T}\;ℒ\left(w_{t},{\dot{w}}_{t}\right)\;dt}.$$

Comparing this dynamic large-deviations principle with (9), we see—not coincidentally—strong similarities. Let us assume that the limit fast fluxes do not influence the concentration, i.e.,

(20) $$\Gamma \tilde{k}\left(\phi [w]\right)=0\;\mathrm{for}\;\mathrm{all}\;w.$$

Naturally, this macroscopic condition entails a sort of decoupling between the slow and fast dynamics, e.g., when the species involved in the slow dynamics act as a catalyst for the fast dynamics. In that case $\rho_{t}^{\left(V\right)}=\phi^{\left(V\right)}\left[W_{t}^{\left(V\right)}\right]$ is exactly the process with generator (3). The contraction principle of large deviations theory (Theorem 4.2.1, [23]) then states that the two large-deviation costs are related via:

$$\int_{0}^{T}\;\hat{ℒ}\left(\rho_{t},{\dot{\rho }}_{t}\right)\;dt=\underset{w_{\left(\cdot \right)}:\rho_{t}=\phi \left[w_{t}\right]}{\inf}\;\int_{0}^{T}\;ℒ\left(w_{t},{\dot{w}}_{t}\right)\;dt.$$

In this setting, this infimum is only with respect to the second variable, because ℒ(w,j) depends on *w* through ϕ[w] only, which in turn arises naturally from the fact that the jump rates depend on the state and not on the integrated flux. Therefore the relation above simplifies further to:

(21) $$\hat{ℒ}\left(\phi [w],s\right)=\underset{j:s=\Gamma j}{\inf}ℒ\left(w,j\right)\;\mathrm{for}\;\mathrm{all}\;w,s,$$

which is consistent with Remark 1. These relations will be the starting point of the general theory that we develop in Section 4. Let us only mention here that as a consequence we also have the relation $\hat{ℋ}\left(\phi [w],\xi \right)=ℋ\left(w,\Gamma^{T}\xi \right)$ for all w,ξ, cf. (7) and (18).

### 3.3. Induced GENERIC Structure

We now investigate whether the large deviations (19) induces some structure in the space of fluxes. It turns out that this is indeed the case. As was found in [12,15] and further studied in [13], under the detailed balance assumption (5) the slow fluxes induce the GGS $\left(R_{+}^{R_{\mathrm{slow}}},\Psi ,F\right)$, where

(22) $$\begin{array}{cc} F(w) & :=\hat{F}\left(\phi [w]\right)=\frac{1}{2}h\left(\rho_{0}+\Gamma w|\rho^{*}\right), \end{array}$$

(23) $$\begin{array}{cc} \Psi^{*}\left(w,\bar{\zeta }\right) & :=\sum_{r\in R_{\mathrm{slow}}}\sigma_{r}\left(w\right)\left(\cosh({\bar{\zeta }}_{r})-1\right)\;\mathrm{and} \end{array}$$

(24) $$\begin{array}{cc} \Psi (w,\bar{j}) & :=\sum_{r\in R_{\mathrm{slow}}}\sigma_{r}\left(w\right)\left(\cosh^{*}\;\left(\frac{{\bar{j}}_{r}}{\sigma_{r}\left(w\right)}\right)+1\right), \end{array}$$

with $\sigma_{r}\left(w\right):=2\sqrt{k_{r,\mathrm{fw}}\left(\phi [w]\right)k_{r,\mathrm{bw}}\left(\phi [w]\right)},r\in R_{\mathrm{slow}}$ and $\cosh^{*}\left(y\right):=\sup_{x\in R}xy-\cosh\left(x\right)=y\arcsin\left(y\right)-\sqrt{1+y^{2}}$. Extending these dissipation potentials to the full flux space by setting $\Psi^{*}\left(w,\zeta \right):=\Psi^{*}\left(w,\bar{\zeta }\right)$ and $\Psi \left(w,j\right):=\Psi \left(w,\bar{j}\right)+\chi_{\left\{\tilde{j}=0\right\}}$, we can decompose the large-deviation cost function as

$$ℒ\left(w,j\right)=\Psi \left(w,j-\tilde{k}\left(\phi \left[w\right]\right)\right)+\Psi^{*}\left(w,-\nabla F(w)\right)+\nabla F\left(w\right)\cdot \left(j-\tilde{k}\left(\phi \left[w\right]\right)\right),$$

and accordingly, the macroscopic evolution (16) as:

$${\dot{w}}_{t}=\nabla_{\zeta }\Psi^{*}\left(w_{t},-\nabla F\left(w_{t}\right)\right)+\tilde{k}\left(\phi \left[w\right]\right).$$

Due to (22) and the decoupling condition (20), the fast fluxes are orthogonal to the driving force, i.e.,

(25) $$\nabla_{w}F\left(w\right)\cdot \tilde{k}\left(\phi \left[w\right]\right)=\nabla_{\rho }\hat{F}\left(\phi [w]\right)\cdot \Gamma \tilde{k}\left(\phi \left[w\right]\right)=0.$$

The quadruple $\left(R_{+}^{R},\;\Psi ,\;F,\;\tilde{k}\circ \phi \right)$ satisfying this condition falls within the class of what is recently coined *pre-(Generalised) GENERIC (pGGEN)* [9,10].

It was shown in those works that non-interaction condition (25) is a necessary and sufficient condition for the existence of an underlying *Generalised GENERIC (GGEN)* structure $\left(R_{+}^{R},\;\Psi ,\;F,\;L,\;E\right)$. (One often needs to introduce an auxiliary energy (e.g., a heat bath) to force conservation of energy, which enlarges the degrees of freedom in the system. To keep notation accessible we ignore this issue). This means that the fast flux term is Hamiltonian $\tilde{k}\left(\phi \left[w\right]\right)=L\left(w\right)\nabla E\left(w\right)$ for some Poisson structure *L* satisfying the Jacobi identity (see Section 4), E is some Hamiltonian energy, and the following two non-interaction conditions are satisfied:

(26) $$\begin{array}{ccc} L(w)\nabla F(w)=0 & \mathrm{and} & \Psi^{*}\left(w,\zeta +z\nabla E(w)\right)=\Psi^{*}\left(w,\zeta \right) \end{array}$$

for all $w\in R_{+}^{R}$, $\zeta \in R^{R}$ and z∈R. These two conditions guarantee that along solutions the free energy is non-increasing and the Hamiltonian energy is conserved:

$$\begin{array}{cc} \frac{d}{dt}F\left(w_{t}\right) & =\underset{\leq 0\;\mathrm{by}\;\mathrm{convexity}}{\underset{︸}{\nabla F\left(w\right)\cdot \nabla_{\zeta }\Psi^{*}\left(w_{t},-\nabla F\left(w\right)\right)}}+\underset{=0\;\mathrm{by}\;(26)}{\underset{︸}{\nabla F(w)\cdot L(w)}}\nabla E\left(w\right)\leq 0,\\ \frac{d}{dt}E\left(w_{t}\right) & =\underset{=0\;\mathrm{by}\;(26)}{\underset{︸}{\nabla E\left(w\right)\cdot \nabla_{\zeta }\Psi^{*}\left(w_{t},-\nabla F\left(w\right)\right)}}+\underset{=0\;\mathrm{by}\;\mathrm{skewsymmetry}}{\underset{︸}{\nabla E(w)\cdot L(w)\nabla E(w)}}=0. \end{array}$$

One main message of this section is that the flux large-deviation cost *ℒ* induces a unique pGGEN system $\left(R_{+}^{R},\;\Psi ,\;F,\;\tilde{k}\circ \phi \right)$. Although this implies the existence of a GGEN system induced by *ℒ*, one can not uniquely decide on the basis of *ℒ* what the “correct” Hamiltonian structure $\left(R_{+}^{R},\;L,\;E\right)$ should be. Additional physical or mathematical arguments needed to uniquely fix the Hamiltonian structure are beyond the scope of this paper; for possible constructions of Poisson structures *L* and energies E, we refer the reader to (Section 4, [10]).

It should be noted that the pGGEN system $\left(R_{+}^{R},\;\Psi ,\;F,\;\tilde{k}\circ \phi \right)$ is rather special in that (25) holds for any $\hat{F}$, since the drift $\tilde{k}\circ \phi$ is “divergence-free”. We will see that such systems play a special role in connecting systems in flux and state space.

Another main message is that the GGS part (22) on flux space is strongly related to the GGS (13) on state space, just like both cost functions are related by (21). These observations will be the basis of the general theory of the next section.

## 4. General Theory

Following the examples of Section 2 and Section 3, we now develop a more abstract framework to study the relation between energy-driven structures in flux and in state space.

### 4.1. Geometry and Notation

**Figure 1:**
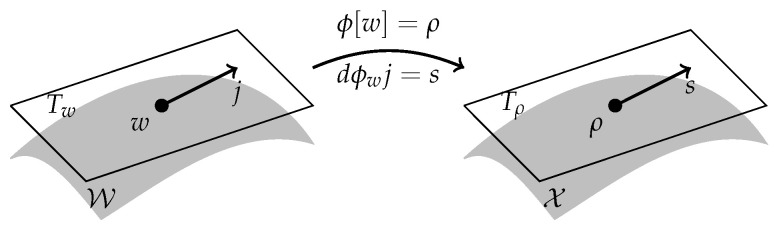
We consider a “flux manifold” W and a “state manifold” X. The “continuity map” ϕ maps points w∈W to points on ρ∈X; its differential $d\phi_{w}$ maps tangents $j\in T_{w}W$ to tangents $s\in T_{\phi }\left[w\right]$.

Throughout this section we assume to be given:

- A differentiable manifold W (“flux space”), where tangents are denoted by (w,j)∈TW and cotangents by $\left(w,\zeta \right)\in T^{*}W$. Note that we distinguish between tangents and cotangents; we write the dual pairing between them as ${}_{T_{w}^{*}}\;{\langle \zeta ,j\rangle }_{T_{w}}$ or simply 〈ζ,j〉;
- A differentiable manifold X (“state space”), where tangents are denoted by (ρ,s)∈TX and cotangents by $\left(\rho ,\xi \right)\in T^{*}X$;
- A surjective differentiable operator ϕ:W→X with bounded linear differential $d\phi_{w}:T_{w}\to T_{\Gamma (w)}$ and adjoint differential $d\phi_{w}^{T}:T_{\phi [w]}^{*}\to T_{w}^{*}$. This defines an abstract continuity equation ϕ[w]=ρ, or in differentiated form $d\phi_{w}j=s$, see Figure 1. Contrary to Section 2 and Section 3, the second continuity equation may now also depend on *w*. In practice, the continuity mapping ϕ[w] depends on the initial state $\rho_{0}$, which we assume to be fixed.
- An L-function $ℒ:TW\to R_{+}$ on flux space (see below for the definition of L-functions). This function could be a dynamic large-deviation cost function corresponding to random fluxes in some microscopic system, but throughout this section it could also be a more general expression.
- An L-function $\hat{ℒ}:TX\to R_{+}$ on state space, related to the flux space L-function via
  (27) $$\hat{ℒ}\left(\rho ,s\right):=\underset{\begin{array}{c} (w,j)\in TW:\\ \phi [w]=\rho ,\;d\phi [w]j=s \end{array}}{\inf}ℒ\left(w,j\right).$$
  This relation is again inspired by large-deviation theory, where such relation holds due to the contraction principle (Theorem 4.2.1, [23]).
- Corresponding to the L-functions are their convex duals with respect to their second variable, i.e., $ℋ:T^{*}W\to R$ and $\hat{ℋ}:T^{*}X\to R$ with
  $$\begin{array}{ccc} ℋ\left(w,\zeta \right):=\underset{j\in T_{w}}{\sup}\langle \zeta ,j\rangle -ℒ\left(w,j\right) & \mathrm{and} & \hat{ℋ}\left(\rho ,\xi \right):=\underset{s\in T_{\rho }}{\sup}\langle \xi ,s\rangle -ℒ\left(\rho ,\xi \right). \end{array}$$
  We express assumptions in terms of these duals, since in practice they are often more explicitly given than their corresponding L-functions.

Recall that in the previous section we saw that ℒ(w,j) depends on *w* through ρ=ϕ[w] only. This condition becomes slightly more complicated in non-flat spaces, for a number of reasons. Firstly, the flux *j* can not be kept fixed while changing *w*, unless one has a path-independent notion of parallel transport, i.e., the space is flat. Secondly, even if ℒ(w,j) would not depend on *w*, this dependence could re-enter through the continuity equation in the infimum $\inf_{j:d\phi_{w}j=s}ℒ\left(w,j\right)$. Therefore, the condition that we need is that $\hat{ℒ}\left(\phi \left[w\right],s\right)=\inf_{\begin{array}{c} j\in T_{w}:\\ d\phi_{w}j=s \end{array}}ℒ\left(w,j\right)$ for all $w\in W,s\in T_{\phi [w]}$. It is easily seen that this condition is equivalent to the following flux invariance:

(28) $$\mathrm{for}\;\mathrm{fixed}\;\left(\rho ,\xi \right)\in T^{*}X,\;\mathrm{the}\;\mathrm{function}\;\phi^{-1}\left[\rho \right]∋w\mapsto ℋ\left(w,d\phi_{w}^{T}\xi \right)\;\mathrm{does}\;\mathrm{not}\;\mathrm{depend}\;\mathrm{on}\;w.$$

All manifolds and functionals are assumed to be sufficiently differentiable wherever needed. For a (differentiable) functional F:W→R (and similarly on flux space) we write $dF\left(w\right)\in T_{w}^{*}$ for the derivative, in the sense that on a curve $\frac{d}{dt}F\left(w_{t}\right)=\langle dF\left(w_{t}\right),{\dot{w}}_{t}\rangle$.

### 4.2. Definitions

We now define the notions of L-functions, dissipation potentials, GGS, pGGEN, Poisson operators and GGEN on flux space; the same concepts on state space are defined analogously. Naturally all notions are compatible with the exposition from Section 2 and Section 3.

**Definition** **1.** *We say $ℒ:TW\to R_{+}$ is an L-function whenever for all w∈W:*

*(i)*  *j↦ℒ(w,j) is convex;*
*(ii)*  *$ℒ\left(w,j\right)=0\Leftrightarrow j=A_{ℒ}\left(w\right)$ for some unique vector field $A_{ℒ}$.*

Since *ℒ* is non-negative, the second condition simply means that *ℒ* should have a unique minimiser; this minimiser corresponds to an evolution equation of the type ${\dot{w}}_{t}=A_{ℒ}\left(w_{t}\right)$. Indeed, since $A_{ℒ}\left(w\right)$ is a minimiser, it will generally satisfy the implicit equation $d_{j}ℒ\left(w,A_{ℒ}\left(w\right)\right)=0$. Due to the convexity, *ℒ* is also the convex dual of *ℋ*.

Central to GGS, pGGEN and GGEN is the notion of dissipation potentials:

**Definition** **2.** *A function $\Psi :TW\to R_{+}$ is called a* dissipation potential *whenever for all w∈W:*

*(i)*  j↦Ψ(w,j) is convex;
*(ii)*  Ψ(w,0)=0;

*If these conditions hold, then the same conditions hold for the (pre-)dual dissipation potential*

(29) $$\Psi^{*}\left(w,\zeta \right):=\underset{j\in T_{w}}{\sup}\langle \zeta ,j\rangle -\Psi \left(w,j\right).$$

*We also say that $\left(\Psi ,\Psi^{*}\right)$ is a* dissipation potential *pair whenever *Ψ* is a dissipation potential.*

**Definition** **3.** *A* generalised gradient system *(GGS) is a triple (W,Ψ,F), where W is a differentiable manifold, F:W→R and $\Psi :TW\to R_{+}$ is a dissipation potential. We say that an L-function ℒ induces a GGS (W,Ψ,F) whenever for all (w,j)∈TW:*

(30) $$ℒ\left(w,j\right)=\Psi \left(w,j\right)+\Psi^{*}\left(w,-dF(w)\right)+\langle dF\left(w\right),j\rangle .$$

As explained in Section 3.3, by extending a GGS with an orthogonal drift one arrives at

**Definition** **4** ([10])**.** *A* Generalised pre-GENERIC system *(pGGEN) is a quadruple (W,Ψ,F,b), where W is a differentiable manifold, *Ψ* is a dissipation potential, F:W→R, and $b\left(w\right)\in T_{w}$ is a vector field such that:*

〈dF(w),b(w)〉=0forallw∈W.

*We say that an L-function induces a pGGEN (W,Ψ,F,b) whenever for all (w,j)∈TW:*

$$ℒ\left(w,j\right)=\Psi \left(w,j-b(w)\right)+\Psi^{*}\left(w,-dF(w)\right)+\langle dF\left(w\right),j\rangle .$$

Finally, if the drift has the form of an Hamiltonian system that behaves more or less independently of the GGS part we arrive at a Generalised GENERIC system. In order to define this we first define:

**Definition** **5.** *A linear operator $L:T^{*}W\to TW$ is called a Poisson structure if it satisfies the Jacobi identity*

$${\left\{{\left\{F_{1},F_{2}\right\}}_{L},F_{3}\right\}}_{L}+{\left\{{\left\{F_{2},F_{3}\right\}}_{L},F_{1}\right\}}_{L}+{\left\{{\left\{F_{3},F_{1}\right\}}_{L},F_{2}\right\}}_{L}=0$$

*for all $F_{1,2,3}:W\to R$, where ${\left\{F_{1},F_{2}\right\}}_{L}\left(w\right):=\langle dF_{1}\left(w\right),L\left(w\right)dF_{2}\left(w\right)\rangle$;*

Jacobi’s identity implies skew symmetry, i.e., $\langle \zeta_{1},L\left(w\right)\zeta_{2}\rangle =-\langle \zeta_{2},L\left(w\right)\zeta_{1}\rangle$ for all w∈W, $\zeta_{1},\zeta_{2}\in T_{w}$; in particular one has 〈ζ,L(w)ζ〉=0. Finally,

**Definition** **6** (Section 2.5, [8])**.** *A* generalised GENERIC system *(GGEN) is a quintuple (W,Ψ,F,L,E), where W is a differentiable manifold, *Ψ* is a dissipation potential, E,F:W→R, $L:T^{*}W\to TW$ is a Poisson structure, and the two non-interaction conditions are satisfied:*

(31) $$\begin{array}{c} L(w)dF(w)=0\;for\;all\;w\in W,\;and \end{array}$$

(32) $$\begin{array}{c} \Psi^{*}\left(w,\zeta +\lambda dE(w)\right)=\Psi^{*}\left(w,\zeta \right)\;for\;all\;\left(w,\zeta \right)\in T^{*}W\;and\;\lambda \in R. \end{array}$$

*We say that an L-function induces a GGEN (W,Ψ,F,L,E) whenever for all (w,j)∈TW:*

(33) $$ℒ\left(w,j\right)=\Psi \left(w,j-L(w)dE(w)\right)+\Psi^{*}\left(w,-dF(w)\right)+\langle dF\left(w\right),j\rangle .$$

**Remark** **2.** *If a GGS, pGGEN or GGEN is given on a manifold W, and the dissipation potentials are quadratic (as explained in Section 2.4), then one can use the positive definite operator $K\left(w\right)\xi :=d_{\zeta }\Psi^{*}\left(w,\zeta \right)$ to define a new manifold, and redefine everything on this manifold. This allows to study the structures from a more geometric point of view.*

### 4.3. From L-Functions to GGS, pGGEN and GGEN

We now recall some of the main results from [4,10], that give necessary and sufficient conditions for an L-function to induce a GGS or pGGEN. A similar result for GGEN does not exist, since an L-function does not uniquely determine a Poisson operator and Hamiltonian energy. However, from a pGGEN one can always construct a GGEN (in a non-unique way); for that result we refer the reader to (Section 4, [10]).

Again, the following results are described, but not restricted to flux space.

**Theorem** **1** (Proposition 2.1 & Theorem 2.1, [4])**.** *Let $ℒ:TW\to R_{+}$ be an L-function with convex dual ℋ, and let F:W→R be given. Then the following statements are equivalent:*

*(i)*  ℒ induces a GGS (W,Ψ,F) for some dissipation potential *Ψ*,
*(ii)*  $ℋ\left(w,\zeta \right)=\Psi^{*}\left(w,\zeta -d_{w}F\left(w\right)\right)-\Psi^{*}\left(w,-dF(w)\right)$ for some dissipation potential $\Psi^{*}$,
*(iii)*  $d_{\zeta }ℋ\left(w,d_{w}F\left(w\right)\right)=0,$             (34)
*(iv)*  $d_{j}ℒ\left(w,0\right)=d_{w}F\left(w\right).$              (35)

*In that case $\Psi^{*}$ (and indirectly *Ψ*) is uniquely determined by*

(36) $$\Psi^{*}\left(w,\zeta \right)=ℋ\left(w,\zeta +d_{w}F\left(w\right)\right)-ℋ\left(w,d_{w}F\left(w\right)\right).$$

From condition (35) we see that F, if it exists, is uniquely given up to constants. Note in particular that conditions (34) and (35) do not involve Ψ.

**Theorem** **2** (Theorem 3.6, [10])**.** *Let $ℒ:TW\to R_{+}$ be an L-function with convex dual ℋ, and let F:W→R and a vector field $b\left(w\right)\in T_{w}$ be given for which $\langle d_{w}F\left(w\right),b\left(w\right)\rangle =0$. Then the following statements are equivalent:*

*(i)*  ℒ induces a pGGEN (W,Ψ,F,b) for some dissipation potential *Ψ*,
*(ii)*  $ℋ\left(w,\zeta \right)=\Psi^{*}\left(w,\zeta -d_{w}F\left(w\right)\right)-\Psi^{*}\left(w,-d_{w}F\left(w\right)\right)+\langle \zeta ,b\left(w\right)\rangle for\;some\;dissipation\;potential\;\Psi^{*},$ (37)
*(iii)*  $d_{\zeta }ℋ\left(w,d_{w}F\left(w\right)\right)=b\left(w\right)$,
*(iv)*  $d_{j}ℒ\left(w,b(w)\right)=d_{w}F\left(w\right).$              (38)

*In that case $\Psi^{*}$ is uniquely determined by*

(39) $$\Psi^{*}\left(w,\zeta \right)=ℋ\left(w,\zeta +d_{w}F\left(w\right)\right)-ℋ\left(w,d_{w}F\left(w\right)\right)-\langle \zeta ,b\left(w\right)\rangle .$$

From condition (37) we see that *ℋ* must consist of a convex part and a linear part 〈ζ,b(w)〉, so that the drift *b* is a priori and uniquely fixed by *ℋ*. Therefore F is again uniquely fixed by condition (38). This is different from the GENERIC setting; LdE uniquely defines dF and vice versa, but the whole quintuple may not be unique. However, one can still state a GENERIC analogue of Theorems 1 and 2 as follows:

**Proposition** **1.** *Let $ℒ:TW\to R_{+}$ be an L-function with convex dual ℋ, and let a Poisson structure $L:T^{*}W\to TW$ and energies E,F:W→R be given such that the non-interaction condition LdF=0 holds. Then the following statements are equivalent:*

*(i)*  *ℒ induces a GGEN (W,Ψ,F,L,E) for some dissipation potential *Ψ*,*
*(ii)*  *$d_{s}ℒ\left(w,L\left(w\right)d_{w}E\left(w\right)\right)=d_{w}F\left(w\right)$ and*
  (40) $$ℒ\left(w,j\right)=\infty \;\;for\;all\;j\in T_{w}\;for\;which\;\langle dE,j\rangle \neq 0,$$
*(iii)*  $d_{\zeta }ℋ\left(w,d_{w}F\left(w\right)\right)=L\left(w\right)d_{w}E\left(w\right)$*and*          (41)
  (42) $$ℋ\left(w,\zeta +\lambda dE(w)\right)=ℋ\left(w,\zeta \right)\;for\;all\;\left(w,\zeta \right)\in T^{*}W\;and\;\lambda \in R.$$

*In that case $\Psi^{*}$ is uniquely determined by*

(43) $$\Psi^{*}\left(w,\zeta \right)=ℋ\left(w,\zeta +d_{w}F\left(w\right)\right)-ℋ\left(w,d_{w}F\left(w\right)\right).$$

**Proof.**  By Lemma 1 (see below), we can apply Theorem 1 to the shifted L-function $\tilde{ℒ}\left(w,j\right):=ℒ\left(w,j+L\left(w\right)d_{w}E\left(w\right)\right)$ and back. This yields the three equivalences, apart from the other non-interaction condition (31). From the explicit formula (43) one finds that the missing non-interaction condition is equivalent to (40) and to (42). ☐

The previous proof made use of the following lemma:

**Lemma** **1.** *Let $L:T^{*}W\to TW$ be a Poisson operator, E,F:W→R be energies and *Ψ* be a dissipation potential such that the non-interaction conditions *(31)* and *(32)* hold. An L-function ℒ induces an GGEN (W,Ψ,F,L,E) if and only if the shifted L-function $\tilde{ℒ}\left(w,j\right):=ℒ\left(w,j+L\left(w\right)d_{w}E\left(w\right)\right)$ induces the GGS (W,Ψ,F).*

**Proof.**  Because of the non-interaction condition (32), the shift transforms relation (33) into (30), and analogously for the other direction. ☐

### 4.4. Relation between Structures in Flux and State Space

We now consider L-functions *ℒ* and $\hat{ℒ}$ on flux and state space, and study how their induced structures are related.

**Proposition** **2.** *Assume that an L-function $ℒ:TW\to R_{+}$ induces a pGGEN (W,Ψ,F,b) where $d\phi_{w}b\left(w\right)=0$ and*

$$\hat{F}\left(\phi [w]\right)=F\left(w\right),\;(up\;to\;constants)$$

*for some $\hat{F}:X\to R$. Then the L-function $\hat{ℒ}$ given by* (27) *induces a GGS $\left(X,\hat{\Psi },\hat{F}\right)$ for some dissipation potential $\hat{\Psi }$.*

**Proof.**  Since $d_{w}\hat{F}\left(\phi \left[w\right]\right)=d\phi_{w}^{T}d_{\rho }\hat{F}\left(\phi \left[w\right]\right)$ and $d\phi_{w}b\left(w\right)=0$, we can rewrite

$$\hat{ℒ}\left(\rho ,s\right)=\underset{w\in W:\phi [w]=\rho }{\inf}\left(\underset{j\in T_{w}:d\phi_{w}j=s}{\inf}\Psi \left(w,j\right)\right)+\Psi^{*}\left(w,-d\phi_{w}^{T}d_{\rho }\hat{F}\left(\rho \right)\right)+\langle d_{\rho }\hat{F}\left(\rho \right),s\rangle ,$$

and because $\Psi ,\Psi^{*}$ is a dissipation potential pair, clearly

$$\hat{ℒ}\left(\rho ,s\right)-\langle d_{\rho }\hat{F}\left(\rho \right),s\rangle \geq \underset{w\in W:\phi [w]=\rho }{\inf}\Psi^{*}\left(w,-d\phi_{w}^{T}d_{\rho }\hat{F}\left(\rho \right)\right)=\hat{ℒ}\left(\rho ,0\right).$$

This is equivalent to $d_{\rho }\hat{F}\left(\rho \right)\in \partial_{s}\hat{ℒ}\left(\rho ,0\right)=\left\{d_{s}\hat{ℒ}\left(\rho ,0\right)\right\}$, which by Theorem 1 implies that $\hat{ℒ}$ induces a GGS $\left(X,\hat{\Psi },\hat{F}\right)$ for some $\hat{\Psi }$. ☐

In the above proposition, F also depends on *w* through ϕ[w] only, which is a very physical assumption. It does imply however, that the equilibria of the flux gradient system can only be unique up to the kernel of ϕ; this kernel can be interpreted as a generalisation of divergence-free vector fields.

A natural question is now whether we can turn the statement of Proposition 2 around. Indeed, if the invariance condition (28) holds and we restrict to pGGEN with “divergence-free drifts”, then the statement becomes an equivalence, and we have an explicit relation between the flux and state dissipation potentials. This is a stronger version of the statement in (Proposition 4.7, [15]), where we related GGS to so-called “force structures”.

**Theorem** **3.** *Assume that an L-function $ℒ:TW\to R_{+}$ with corresponding dual ℋ satisfies the invariance condition *(28)*, and let the L-function $\hat{ℒ}:X\to R_{+}$ be given by *(27)*. Then the following statements are equivalent:*

*(i)*  *ℒ induces a pGGEN (W,Ψ,F,b) with $d\phi_{w}b\left(w\right)=0$ and $\hat{F}\circ \phi$ for some $\hat{F}:X\to R$,*
*(ii)*  *$\hat{ℒ}$ induces a GGS $\left(X,\;{\hat{\Psi }}^{*},\;\hat{F}\right)$.*

*If these statement hold, then the dissipation potentials $\hat{\Psi }$ and ${\hat{\Psi }}^{*}$ are related to *Ψ* and $\Psi^{*}$ through*

(44) $$\begin{array}{ccc} \hat{\Psi }\left(\phi [w],s\right)=\underset{\begin{array}{c} j\in T_{w}:\\ d\phi_{w}j=s \end{array}}{\inf}\Psi \left(w,j\right) & \mathrm{and}\;\mathrm{respectively} & {\hat{\Psi }}^{*}\left(\phi [w],\xi \right)=\Psi^{*}\left(w,d\phi_{w}^{T}\xi \right). \end{array}$$

**Proof.**  Assume that *ℒ* induces a pGGEN (W,Ψ,F,b) with $d\phi_{w}b\left(w\right)=0$ and $\hat{F}\circ \phi$. Since by assumption $ℋ(w,\;d\phi_{w}^{T}\xi )$ does not depend on $w\in \phi^{-1}\left[\rho \right]$, by (36) the expression $\Psi^{*}\left(w,\;d\phi_{w}^{T}\xi \right)$ is also invariant under this choice. Therefore we can define ${\hat{\Psi }}^{*}\left(\rho ,\xi \right)$ by (44); it is easily checked that its convex dual is given by (44). We can write:

$$\begin{array}{cc} \hat{ℒ}\left(\rho ,s\right) & =\underset{d\phi_{w}j=s}{\inf}\left\{\Psi \left(w,j-b(w)\right)+\Psi^{*}\left(w,-d_{w}F\left(w\right)\right)+\langle d_{w}F\left(w\right),j\rangle \right\}\\ & =\underset{d\phi_{w}j=s}{\inf}\Psi \left(w,j\right)+\Psi^{*}\left(w,-d\phi_{w}^{T}d_{\rho }\hat{F}\left(\rho \right)\right)+\langle d_{\rho }\hat{F}\left(\rho \right),s\rangle \\ & =\hat{\Psi }\left(\rho ,s\right)+{\hat{\Psi }}^{*}\left(\rho ,-d_{\rho }\hat{F}\left(\rho \right)\right)+\langle d_{\rho }\hat{F}\left(\rho \right),s\rangle , \end{array}$$

and hence $\hat{ℒ}$ induces the GGS $\left(X,\hat{\Psi },\hat{F}\right)$, which is unique by Theorem (1).

For the other direction, assume that $\hat{ℒ}$ induces a GGS $\left(X,{\hat{\Psi }}^{*},\hat{F}\right)$. Define $b\left(w\right):=d_{\zeta }ℋ\left(w,d_{w}F\left(w\right)\right)$. Then by the invariance condition $ℋ\left(w,d\phi_{w}^{T}\xi \right)=\hat{ℋ}\left(\rho ,\xi \right)$ and by (34):

$$d\phi_{w}b\left(w\right)=d\phi_{w}d_{\zeta }ℋ\left(w,d_{w}F\left(w\right)\right)=d\phi_{w}d_{\zeta }ℋ\left(w,d\phi_{w}d_{\rho }\hat{F}\left(\rho \right)\right)=d_{\xi }ℋ\left(\rho ,d_{\rho }\hat{F}\left(\rho \right)\right)=0.$$

Now define $\Psi^{*}$ by (39). In particular $\Psi^{*}\left(w,-dF(w)\right)=-ℋ\left(w,d_{w}F\left(w\right)\right)$ since $\langle d_{w}F\left(w\right),b\left(w\right)\rangle =0$ and, by the definition of L-functions, ℋ(w,0)=0. Then (37) holds and hence by Theorem 2 the flux L-function *ℒ* induces the pGGEN (W,Ψ,F,b). ☐

Due to the non-uniqueness of induced GGEN systems, there is no similar “if and only if” statement for the GENERIC setting. Nevertheless, in one direction, the GGEN analogue of Theorem 3 is:

**Proposition** **3.** *Assume that an L-function ℒ:TW→R induces a GGEN (W,Ψ,F,L,E) where*

(45) $$\begin{array}{ccc} \hat{F}\left(\phi [w]\right)=F\left(w\right) & and & \hat{E}\left(\phi [w]\right)=E\left(w\right),\;(up\;to\;constants) \end{array}$$

*for some $\hat{F},\hat{E}:X\to R$, and that*

(46) $$d\phi_{w}L\left(w\right)d\phi_{w}^{T}=:\hat{L}\left(\rho \right)\;depends\;on\;w\;through\;\rho =\phi [w]\;only.$$

*Then the L-function $\hat{ℒ}\left(\rho ,s\right)$ given by *(27)* induces a GGEN $\left(X,\hat{\Psi },\hat{F},\hat{L},\hat{E}\right)$ for some dissipation potential $\hat{\Psi }$.*

*If in addition, ℋ satisfies the invariance principle *(28)*, then $\hat{\Psi }$ and ${\hat{\Psi }}^{*}$ are related to *Ψ* and $\Psi^{*}$ through *(44)*.*

**Proof.**  We again apply Lemma 1 to transform the problem into a problem of GGSs. Indeed, the L-function $\tilde{ℒ}\left(w,j\right):=ℒ\left(w,j+L\left(w\right)d_{w}E\left(w\right)\right)$ induces the GGS (W,Ψ,F). Hence by Proposition 2, a GGS $\left(X,\hat{\Psi },\hat{F}\right)$ (for some $\hat{\Psi }$) is induced by the L-function

$$\hat{\tilde{ℒ}}\left(\rho ,s\right):=\underset{\phi [w]=\rho }{\inf}\;\underset{d\phi_{w}j=s}{\inf}\tilde{ℒ}\left(w,j\right)=\underset{\phi [w]=\rho }{\inf}\;\underset{d\phi_{w}\left(j-L\left(w\right)d_{w}E\left(w\right)\right)=s}{\inf}ℒ\left(w,j\right)=\hat{ℒ}\left(\rho ,s+\hat{L}\left(\rho \right)d_{\rho }E\left(\rho \right)\right).$$

If we can now validate that $\hat{L}$ is a Poisson structure, and that the non-interaction conditions are satisfied for $\left(X,\hat{\Psi },\hat{F},\hat{L},\hat{E}\right)$, then Lemma 1 concludes the proof.

For the Poisson structure, note that, for any smooth ${\hat{F}}_{1},{\hat{F}}_{2}:X\to R$, the Lie bracket remains unaltered:

$$\begin{array}{cc} {\left\{{\hat{F}}_{1},{\hat{F}}_{2}\right\}}_{\hat{L}}\left(\phi [w]\right) & ={\left\{F_{1}\circ \phi ,F_{2}\circ \phi \right\}}_{L}\left(w\right). \end{array}$$

The non-interaction condition (32) is clearly satisfied as for any w∈W we have

$$\hat{L}\left(\phi [w]\right)d_{\rho }\hat{F}\left(\phi [w]\right)=d\phi_{w}L\left(w\right)d\phi_{w}^{T}d_{\rho }\hat{F}\left(\phi [w]\right)=d\phi_{w}L\left(w\right)d_{w}F\left(w\right)=0.$$

To check the other non-interaction condition (31) we use the equivalent formulation (42). Indeed, for any $\left(\rho ,\xi \right)\in T^{*}X$ and λ∈R,

$$\begin{array}{c} \hat{ℋ}\left(\rho ,\xi +\lambda d_{\rho }d\hat{E}\left(\rho \right)\right)=\underset{\phi [w]=\rho }{\sup}ℋ\left(w,d\phi_{w}^{T}(\xi +\lambda d_{\rho }\hat{E}\left(\phi \left[w\right]\right)\right))\\ =\underset{\phi [w]=\rho }{\sup}ℋ\left(w,d\phi_{w}^{T}\xi +\lambda d_{w}E\left(w\right)\right)=\underset{\phi [w]=\rho }{\sup}ℋ\left(w,d\phi_{w}^{T}\xi \right)=\hat{ℋ}(\rho ,\xi ). \end{array}$$

Finally, if *ℋ* satisfies the invariance property (28), then Proposition 2 yields relations (44). ☐

Condition (46) is in a sense a natural one as the following result shows:

**Proposition** **4.** *Assume that L-functions ℒ and $\hat{ℒ}$ induce two GGENs (W,Ψ,F,L,E) and $\left(X,\hat{\Psi },\hat{F},\hat{L},\hat{E}\right)$, where F,E are related to $\hat{F},\hat{E}$ by *(45)*, and ℋ satisfies the invariance property *(28)*. Then*

$$\hat{L}\left(\phi [w]\right)d_{\rho }\hat{E}\left(\phi [w]\right)=d\phi_{w}L\left(w\right)d\phi_{w}^{T}d_{\rho }\hat{E}\left(\phi [w]\right).$$

**Proof.**  For any w∈W and ρ=ϕ[w], we may write $\hat{ℋ}\left(\rho ,\xi \right)=ℋ\left(w,d\phi_{w}^{T}\xi \right)$, and so by (41),

$$\hat{L}\left(\rho \right)d_{\rho }\hat{E}\left(\rho \right)=d_{\xi }\hat{ℋ}\left(\rho ,-d_{\rho }\hat{F}\left(\rho \right)\right)=d\phi_{w}d_{\zeta }ℋ\left(w,-d\phi_{w}d_{\rho }\hat{F}\left(\phi \left[w\right]\right)\right)=d\phi_{w}L\left(w\right)d\phi_{w}d_{\rho }\hat{E}\left(\rho \right).$$

 ☐

## 5. Diffusion

In this section we apply the ideas of the previous section to a model for diffusion. The flux structure related to diffusion is interesting in its own right, and as far as the author is aware, previously unknown. In the next section we show how this model can be coupled with the results of Section 3 to obtain flux and state GGSs/pGGENs for reaction-diffusion systems.

Typical microscopic models of diffusion consist of Brownian particles, or discretised versions thereof, like random walkers or an exclusion process. Since empirical fluxes are a bit easier to define on a lattice, we focus on independent random walkers (With the scaling that we use, the system of independent random walkers is “exponentially equivalent” to a system of Brownian motions, meaning they share the same hydrodynamic limit and large deviations). The model, its many-particle limit and large deviations are similar to e.g., [24,25].

### 5.1. Diffusing Particle System

This microscopic particle system actually has two scaling parameters: the number of particles, which we denote by *V* for consistency with the rest of this paper, and the lattice spacing $\epsilon_{V}$. The speed with which $\epsilon_{V}\to 0$ as V→∞ is irrelevant. For fixed *V*, let ${\left(X_{t,i}\right)}_{i=1}^{V}$ be independent random walkers on the lattice ${\left(\epsilon_{V}Z\right)}^{d}$ with jump rate $\epsilon^{-2}$. Define the random concentration and (integrated, net) flux by:

$$\begin{array}{cc} \rho_{t}^{\left(V\right)}\left(dx\right) & :=\frac{1}{V}\#\{i=1,\cdots ,V:X_{t,i}\in dx\},\;\mathrm{and}\\ {\bar{W}}_{t,l}^{\left(V\right)}\left(dx\right) & :=\frac{\epsilon_{V}}{V}\#\left\{\mathrm{jumps}\;\tilde{x}\;\mathrm{to}\;\tilde{x}+\epsilon_{V}{𝟙}_{l}\;\mathrm{occurred}\;\mathrm{in}\;\left(0,t\right):\tilde{x}+\frac{1}{2}\epsilon_{V}{𝟙}_{l}\in dx\right\}\\ & \;-\frac{\epsilon_{V}}{V}\#\left\{\mathrm{jumps}\;\tilde{x}+\epsilon_{V}{𝟙}_{l}\;\mathrm{to}\;\tilde{x}\;\mathrm{occurred}\;\mathrm{in}\;\left(0,t\right):\tilde{x}+\frac{1}{2}\epsilon_{V}{𝟙}_{l}\in dx\right\}. \end{array}$$

As usual dx denotes a spatial area, possibly a small box surrounding one lattice site, and $\rho_{t}^{\left(V\right)}$ and ${\bar{W}}_{t,l}^{\left(V\right)}$ are measures.

**Figure 2:**
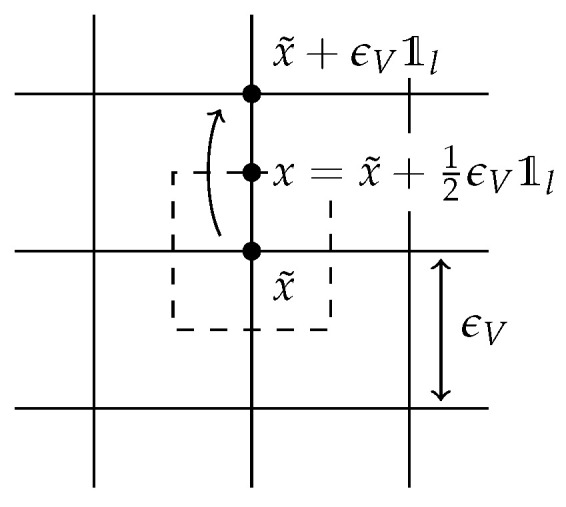
When a particle jumps from $\tilde{x}$ in the direction with unit vector ${𝟙}_{l}$, this event is recorded in the integrated flux ${\bar{W}}_{t,l}$ in the midpoint $x=\tilde{x}+\frac{1}{2}\epsilon_{V}{𝟙}_{l}$. As such the quantity ${\bar{W}}_{t,l}$ measures the net number of particles that have passed through the (upper-right) boundary of an imaginary box around midpoint *x*, drawn in dotted lines.

Now $\rho_{t}^{\left(V\right)}\left(dx\right)$ measures the number of particles present in (lattice points in) an area dx, while ${\bar{W}}_{t,l}^{\left(V\right)}\left(dx\right)$ measures the *net* number of particles that have jumped through all midpoints in dx, in direction ${𝟙}_{l}$, for l=1,⋯,d, see Figure 2. Note that both $\rho^{\left(V\right)}$ and ${\bar{W}}^{\left(V\right)}$ are defined as measures on the lattice with shrinking distance $\epsilon_{V}$ between lattice points; this measure-valued formulation is needed to pass to a continuum limit later on.

The concentrations and fluxes are related by the *V*-dependent continuity equation:

(47) $$\begin{array}{cc} \rho_{t}^{\left(V\right)}\left(dx\right) & =\phi^{\left(V\right)}\left[{\bar{W}}_{t}^{\left(V\right)}\right]\left(dx\right):=\left(\rho_{0}^{\left(V\right)}-{\mathrm{div}}^{\left(\epsilon_{V}\right)}{\bar{W}}_{t}^{\left(V\right)}\right)\left(dx\right)\\ & :=\rho_{0}^{\left(V\right)}\left(dx\right)-\frac{1}{\epsilon_{V}}\sum_{l=1}^{d}\left[{\bar{W}}_{t,l}\left(dx+\frac{1}{2}\epsilon +V{𝟙}_{l}\right)-{\bar{W}}_{t,l}\left(dx-\frac{1}{2}\epsilon_{V}{𝟙}_{l}\right)\right]. \end{array}$$

Using that ${\left(\frac{1}{2}\epsilon_{V}+\epsilon_{V}Z\right)}^{d}\subset R^{d}$, the integrated flux ${\bar{W}}_{t}^{\left(V\right)}$ is a Markov process in $M(R^{d})$ with generator

(48) $$\left(Q^{\left(V\right)}f\right)\left(\bar{w}\right):=\frac{V}{\epsilon_{V}^{2}}\;\int \;\phi^{\left(V\right)}\left[\bar{w}\right]\left(dx\right)\sum_{l=1}^{d}\left(f\left(\bar{w}-\frac{\epsilon_{V}}{V}\delta_{x-\left(\epsilon_{V}/2\right){𝟙}_{l}}\right)-2f\left(\bar{w}\right)+f\left(\bar{w}+\frac{\epsilon_{V}}{V}\delta_{x+\left(\epsilon_{V}/2\right){𝟙}_{l}}\right)\right).$$

Here, the factor $V/\epsilon_{V}^{2}$ comes from the time scaling $\epsilon_{V}^{2}$, together with the fact that we have Vρ(dx) independent particles to choose from.

### 5.2. Limit and Large Deviations

For a test function $f\in C_{b}^{1}\left(M(R^{d})\right)$, we set:

$$\begin{array}{cc} df{\left(\bar{w}\right)}_{l}\left(x\right) & :=\lim_{\tau \to 0}\frac{f\left(\bar{w}+\tau \delta_{x}e_{l}\right)-f\left(\bar{w}\right)}{\tau }. \end{array}$$

The continuity Equation (47) converges to the limit continuity equation (with the usual divergence operator):

(49) $$\begin{array}{cc} \rho_{t}\left(dx\right)=\phi \left[{\bar{w}}_{t}\right]\left(dx\right) & :=\rho_{0}\left(dx\right)-{\mathrm{div}}_{x}{\bar{w}}_{t}\left(dx\right). \end{array}$$

With this notation, as V→∞ the generator (48) converges to (if $\phi \left[\bar{w}\right]\left(dx\right)=\phi \left[\bar{w}\right]\left(x\right)\;dx$):

$$\begin{array}{cc} \left(Q^{\left(\infty \right)}f\right)\left(\bar{w}\right) & :=\int \;{\mathrm{div}}_{x}df\left(\bar{w}\right)\left(x\right)\;\phi \left[\bar{w}\right]\left(dx\right)=-\int \;df\left(\bar{w}\right)\left(x\right)\cdot \nabla_{x}\phi \left[\bar{w}\right]\left(x\right)\;dx. \end{array}$$

As in Section 2.3, the limit generator depends on derivatives of the test function only, and so the process ${\bar{W}}_{t}^{\left(V\right)}$ converges (pathwise in probability) to the deterministic path satisfying Fick’s Law:

$${\dot{\bar{w}}}_{t}=-\nabla_{x}\phi \left[{\bar{w}}_{t}\right].$$

Naturally, combining this equation with the continuity Equation (49) yields the diffusion equation for the empirical measure:

$${\dot{\rho }}_{t}=\frac{d}{dt}\phi \left[{\bar{w}}_{t}\right]=d\phi_{{\bar{w}}_{t}}{\dot{\bar{w}}}_{t}=\Delta_{x}\phi \left[{\bar{w}}_{t}\right]=\Delta_{x}\rho_{t}.$$

Similarly, we derive the dynamic large deviations by studying the non-linear generator:

(50) $$\begin{array}{cc} \left({ℋ}^{\left(V\right)}f\right)\left(\bar{w}\right) & :=\frac{1}{V}e^{-Vf(\bar{w})}\left(Q^{\left(V\right)}e^{Vf}\right)\left(\bar{w}\right)\\ & =\sum_{l=1}^{d}\int \;\frac{e^{Vf\left(\bar{w}-\frac{\epsilon_{V}}{V}\delta_{x-\left(\epsilon_{V}/2\right){𝟙}_{l}}\right)-Vf\left(\bar{w}\right)}-2+e^{Vf\left(\bar{w}+\frac{\epsilon_{V}}{V}\delta_{x+(\epsilon_{V}/2}{𝟙}_{l}\right)-Vf\left(w\right)}}{\epsilon_{V}^{2}}\;\phi^{\left(V\right)}\left[\bar{w}\right]\left(dx\right)\\ & \overset{V\to \infty }{\to }\int \;\left({\mathrm{div}}_{x}df\left(\bar{w}\right)\left(x\right)+{\left|df\left(\bar{w}\right)\left(x\right)\right|}^{2}\right)\;\phi \left[\bar{w}\right]\left(dx\right), \end{array}$$

which follows from expanding the exponentials (with order-$\epsilon_{V}$ exponents) up to second order. Then the following large-deviation principle on flux space holds:

$$\begin{array}{c} \;{\mathrm{Prob}}^{\left(V\right)}\;\left({\bar{W}}_{\left(\cdot \right)}^{\left(V\right)}\approx {\bar{w}}_{\left(\cdot \right)}\right)\overset{V\to \infty }{∼}e^{-V\int_{0}^{T}\;ℒ\left({\bar{w}}_{t},{\dot{\bar{w}}}_{t}\right)\;dt}, \end{array}$$

with

(51) $$\begin{array}{cc} ℋ(\bar{w},\bar{\zeta }) & :=\int \;\left({\mathrm{div}}_{x}\bar{\zeta }\left(x\right)+{\left|\bar{\zeta }\left(x\right)\right|}^{2}\right)\phi \left[\bar{w}\right]\left(dx\right)={∥\bar{\zeta }∥}_{L^{2}\left(\phi \left[\bar{w}\right]\right)}^{2}-\langle \bar{\zeta },\nabla_{x}\phi \left[\bar{w}\right]\rangle ,\;\mathrm{and} \end{array}$$

$$\begin{array}{cc} ℒ(\bar{w},\bar{j}) & :=\underset{\bar{\zeta }}{\sup}\;\langle \bar{\zeta },\bar{j}\rangle -ℋ\left(\bar{w},\bar{\zeta }\right)=\frac{1}{4}{∥\bar{j}+\nabla_{x}\phi \left[\bar{w}\right]∥}_{L^{2}\left(1/\phi \left[\bar{w}\right]\right)}^{2}. \end{array}$$

Note that $d\phi_{\bar{w}}=-{\mathrm{div}}_{x}$ is independent of $\bar{w}$, and by (51), the invariance condition (28) is satisfied. Hence by the contraction principle (Theorem 4.2.1, [23]), one obtains the large-deviation principle corresponding to the states (empirical measures):

$$\begin{array}{c} \;{\mathrm{Prob}}^{\left(V\right)}\;\left(\rho_{\left(\cdot \right)}^{\left(V\right)}\approx \rho_{\left(\cdot \right)}\right)\overset{V\to \infty }{∼}e^{-V\int_{0}^{T}\;\hat{ℒ}\left(\rho_{t},{\dot{\rho }}_{t}\right)\;dt}, \end{array}$$

with

(52) $$\begin{array}{c} \hat{ℒ}\left(\phi \left[\bar{w}\right],s\right):=\underset{\begin{array}{c} \bar{j}\in T_{\bar{w}}:\;-\;{\mathrm{div}}_{x}\bar{j}=s \end{array}}{\inf}ℒ\left(\bar{w},\bar{j}\right)=\frac{1}{4}{∥s-\Delta_{x}\phi \left[\bar{w}\right]∥}_{{\overset{˚}{H}}^{-1}\left(\phi \left[\bar{w}\right]\right)}^{2}, \end{array}$$

using the notation ${∥s∥}_{{\overset{˚}{H}}^{-1}\left(\rho \right)}^{2}:=\sup_{\xi }2s\cdot \xi -{∥\xi ∥}_{{\overset{˚}{H}}^{1}\left(\rho \right)}^{2}:=\sup_{\xi }2s\cdot \xi -{∥\nabla_{x}\xi ∥}_{L^{2}\left(\rho \right)}^{2}$.

### 5.3. Induced GGSs in Flux and State Space

We can now apply Theorem 1 to extract a GGS from the L-function *ℒ*. We first choose the ‘naive’ flat manifold of non-negative vector measures $W:=M_{+}\left(R^{d};R^{d}\right)$ (equipped with the flat total variation metric). It is easily checked that condition (34) holds for the free energy given by:

(53) $$F\left(\bar{w}\right):=\frac{1}{2}\int \;\phi \left[\bar{w}\right]\left(dx\right)\log\phi \left[\bar{w}\right]\left(x\right)-\phi \left[\bar{w}\right]\left(dx\right),$$

where we identify $\phi \left[\bar{w}\right]\left(dx\right)=\phi \left[\bar{w}\right]\left(x\right)\;dx$, and we implicitly set F(w)=∞ whenever the measure is not absolutely continuous. (This expression can again be seen as a relative entropy, cf. (22), but now with respect to the Lebesgue measure, where the measure of the whole space—in this case infinity—is omitted. See also (Proposition 3.2, [4]) for a general result in locally finite measure spaces). The dissipation potentials are obtained from (36) and (29), which yields

(54) $$\begin{array}{ccc} \Psi^{*}\left(\bar{w},\bar{\zeta }\right):={∥\bar{\zeta }∥}_{L^{2}\left(\phi \left[\bar{w}\right]\right)}^{2} & \mathrm{and} & \Psi \left(\bar{w},\bar{j}\right):=\frac{1}{4}{∥\bar{j}∥}_{L^{2}\left(1/\phi \left[\bar{w}\right]\right)}^{2}. \end{array}$$

Theorem 1 states that *ℒ* induces the GGS $\left(M_{+}\left(R^{d}\right),\Psi ,F\right)$ on flux space.

For the state space, it is well-known that the state L-function (52) induces the entropy-Wasserstein gradient flow of the entropy functional [4,26,27,28]. By the theory developed in Section 4, we can now see how this gradient structure is related to the flux gradient structure. Indeed, the flux free energy F depends on state only, i.e., $F\left(\bar{w}\right)=\hat{F}\left(\phi \left[\bar{w}\right]\right))$, where $\hat{F}\left(\rho \right)=\frac{1}{2}\int \;\rho \left(dx\right)\log\rho \left(x\right)-\rho \left(dx\right)$, and so by Proposition 2 the state L-function $\hat{ℒ}$ induces a GGS driven by $\hat{F}$. Moreover, since the invariance condition (28) holds, the dissipation potentials are related by Equations (44) (recall the norms introduced above in (52)):

$$\begin{array}{ccc} {\hat{\Psi }}^{*}\left(\rho ,\xi \right)={∥\nabla_{x}\xi ∥}_{L^{2}\left(\rho \right)}^{2}=:{∥\xi ∥}_{{\overset{˚}{H}}^{1}\left(\rho \right)}^{2}, & \mathrm{and} & \hat{\Psi }\left(\rho ,s\right)=\underset{-{\mathrm{div}}_{x}\bar{j}=s}{\inf}\frac{1}{4}{∥\bar{j}∥}_{L^{2}\left(1/\rho \right)}^{2}=:\frac{1}{4}{∥s∥}_{{\overset{˚}{H}}^{-1}\left(\rho \right)}^{2}. \end{array}$$

### 5.4. A New Geometry

The form of the dissipation potential Ψ and $\hat{\Psi }$ suggests that it is more natural to use different manifolds in the spirit of Remark 2. For the state space this points to the space $W:=P_{2}\left(R^{d}\right)=\left\{\rho \in P\left(R^{d}\right):\int \;x^{2}\;\rho \left(dx\right)<\infty \right\}$ of probability measures of finite second moment space, equipped with the Monge–Kantorovich–Wasserstein metric [29]:

$$d_{P_{2}}{\left(\rho_{0},\rho_{1}\right)}^{2}:=\underset{\begin{array}{c} \gamma \in P(R^{d}\times R^{d}):\\ \gamma \left(\cdot \times R^{d}\right)=\rho_{0}\left(\cdot \right)\\ \gamma \left(R^{d}\times \cdot \right)=\rho_{1}\left(\cdot \right) \end{array}}{\inf}\;{\;\int \int \;}_{R^{d}\times R^{d}}{\left|x-y\right|}^{2}\;\gamma \left(dx\;dy\right).$$

with tangent and cotangent space $T_{\rho }={\overset{˚}{H}}^{-1}\left(\rho \right)$ and $T_{\rho }^{*}={\overset{˚}{H}}^{1}\left(\rho \right)$. For this setting, the inverse metric tensor $K_{P_{2}}\left(\rho \right):T_{\rho }^{*}\to T_{\rho }$ is known by the Benamou–Brenier formula (Theorem 8.1, [29]) to be $K_{P_{2}}\left(\rho \right)\xi :=-2{\mathrm{div}}_{x}\rho \nabla_{x}\xi$, so that the GGS is indeed the entropy-Wasserstein gradient flow [30]:

$$\Delta_{x}\rho_{t}={\dot{\rho }}_{t}=d_{\xi }{\hat{\Psi }}^{*}\left(\rho_{t},-d\hat{F}\left(\rho_{t}\right)\right)=-2K_{P_{2}}\left(\rho_{t}\right)d\hat{F}\left(\rho_{t}\right)=:-2{\mathrm{grad}}_{\rho_{t}}\hat{F}\left(\rho_{t}\right).$$

Motivated by this observation we can take for the flux manifold the space of signed vector measures of finite *first* moment $M_{1}\left(R^{d};R^{d}\right):=\left\{\bar{w}\in M\left(R^{d};R^{d}\right):\int \;\left|x\right|\;\left|\bar{w}\right|\left(dx\right)<\infty \right\}$. This choice guarantees that the corresponding states have finite second moment (once $\rho_{0}\in P_{2}\left(R^{d}\right)$):

$$\int \;{\left|x\right|}^{2}\;\rho \left(dx\right)=\int \;{\left|x\right|}^{2}\;\rho_{0}\left(dx\right)+\int \;x\cdot \bar{w}\left(dx\right)-\mathrm{non}-\mathrm{neg}.\mathrm{bnd}.\mathrm{term}.<\infty .$$

Moreover, we can now use the dissipation potential to construct a natural metric on Y:

(55) $$d_{M_{1}}{\left({\bar{w}}_{0},{\bar{w}}_{1}\right)}^{2}:=\underset{\begin{array}{c} \tilde{w}:\left(0,1\right)\to W:\\ {\tilde{w}}_{0}={\bar{w}}_{0},\;{\tilde{w}}_{1}={\bar{w}}_{1} \end{array}}{\inf}\int_{0}^{1}\;{∥{\dot{\tilde{w}}}_{t}∥}_{L^{2}\left(1/\left(\rho_{0}-{\mathrm{div}}_{x}{\tilde{w}}_{t}\right)\right)}^{2}\;dt,$$

where the infimum runs over paths of fluxes for which $\rho_{0}-\mathrm{div}{\tilde{w}}_{t}$ remains non-negative. The corresponding tangent and cotangent spaces (in the interior of the domain) are simply $T_{\bar{w}}=L^{2}\left(1/\left(\rho_{0}-{\mathrm{div}}_{x}\bar{w}\right)\right)$ and $T_{\bar{w}}^{*}=L^{2}\left(\rho_{0}-{\mathrm{div}}_{x}\bar{w}\right)$ and the inverse metric tensor $K_{M_{1}}\left(\bar{w}\right):T_{\bar{w}}^{*}\to T_{\bar{w}}$ is $K_{M_{1}}\left(\bar{w}\right)\bar{\zeta }=\bar{\zeta }/\left(\rho_{0}-{\mathrm{div}}_{x}\bar{w}\right)$. This yields an interesting geometry in flux space, which, as far as the author is aware, is still unknown in the literature.

## 6. A Simple Reaction-Diffusion Model

We now combine the models from Section 3 and Section 5 to study reaction-diffusion models in flux and state space. The stochastic particle system will now consist of ‘reacting random walkers’. It is known that, if the reaction networks include reactions of different orders (unimolecular, bimolecular, etc.) and we only allow particles to react if the required number of particles are present within the same site/compartment, then the model may not converge to the expected reaction-diffusion Equation [31]. The reason behind this is that for a multimolecular reaction, it becomes very unlikely that the required amount of reactants are all within one site/compartment; different order reactions would require different scalings. This is beyond the scope of the current paper. However we can already illustrate the combination of reaction and transport fluxes for a simple system of unimolecular equations of the type:

$$\begin{array}{ccc} A\overset{\kappa_{\mathrm{fw}}}{\to }B & \mathrm{and} & B\overset{\kappa_{\mathrm{bw}}}{\to }A. \end{array}$$

In this section we consider GGSs only, hence we shall always consider net rather than one-way fluxes.

### 6.1. Reacting and Diffusing Particle System

Since we consider unimolecular reactions only, we can take *independent* reacting random walkers on the scaled lattice ${\left(\epsilon_{V}Z\right)}^{d}$, where each reaction occurs locally at each lattice site with rate $\kappa_{\mathrm{fw}}$ or $\kappa_{\mathrm{bw}}$ respectively, so that

$$\begin{array}{ccc} \frac{1}{V}\lambda_{\mathrm{fw}}^{\left(V\right)}\left(\rho \left(dx\right)\right)\equiv \kappa_{\mathrm{fw}}\rho_{A}\left(dx\right) & \mathrm{and} & \frac{1}{V}\lambda_{\mathrm{bw}}^{\left(V\right)}\left(\rho \left(dx\right)\right)\equiv \kappa_{\mathrm{bw}}\rho_{B}\left(dx\right). \end{array}$$

For the transport mechanism, we assume that the two species A,B hop to neighbouring lattice sites with rates $D_{A}$ and $D_{B}$ respectively.

As before we consider the random concentrations, as well as the integrated (net) fluxes, where we now distinguish between transport fluxes and reaction fluxes. If $X_{t,i}\in \left(\epsilon_{V}Z^{d}\right)\subset R^{d}$ is the position and $Y_{t,i}\in Y=\left\{A,B\right\}$ is the species of the *i*-th particle, then

$$\begin{array}{cc} \rho_{t,y}^{\left(V\right)}\left(dx\right) & :=\frac{1}{V}\#\{i=1,\cdots ,V:X_{t,i}\in dx\;\mathrm{and}\;Y_{t,i}=y\},\\ {\bar{W}}_{t,\mathrm{tr},y,l}^{\left(V\right)}\left(dx\right) & :=\frac{\epsilon_{V}}{V}\#\left\{\mathrm{jumps}\;\tilde{x}\;\mathrm{to}\;\tilde{x}+\epsilon_{V}{𝟙}_{l}\;\mathrm{of}\;\mathrm{species}\;y\;\mathrm{occurred}\;\mathrm{in}\;\left(0,t\right):\tilde{x}+\frac{1}{2}\epsilon_{V}{𝟙}_{l}\in dx\right\}\\ & \;-\frac{\epsilon_{V}}{V}\#\left\{\mathrm{jumps}\;\tilde{x}+\epsilon_{V}{𝟙}_{l}\;\mathrm{to}\;\tilde{x}\;\mathrm{of}\;\mathrm{species}\;y\;\mathrm{occurred}\;\mathrm{in}\;\left(0,t\right):\tilde{x}+\frac{1}{2}\epsilon_{V}{𝟙}_{l}\in dx\right\},\\ {\bar{W}}_{t,\mathrm{re}}^{\left(V\right)}\left(dx\right) & :=\frac{1}{V}\#\{\mathrm{forward}\;\mathrm{reactions}\;r\;\mathrm{occurred}\;\mathrm{in}\;(0,t)\;\mathrm{and}\;\mathrm{in}\;\mathrm{area}\;dx\},\\ & \;-\frac{1}{V}\#\left\{\mathrm{backward}\;\mathrm{reactions}\;r\;\mathrm{occurred}\;\mathrm{in}\;(0,t)\;\mathrm{and}\;\mathrm{in}\;\mathrm{area}\;dx\right\}. \end{array}$$

The concentrations and fluxes are again related by a continuity equation:

(56) $$\begin{array}{cc} \rho_{t,y}^{\left(V\right)}\left(dx\right) & =\phi^{\left(V\right)}{\left[{\bar{W}}_{t}^{\left(V\right)}\right]}_{y}\left(dx\right):={\left(\rho_{0}^{\left(V\right)}-{\mathrm{div}}^{\left(\epsilon_{V}\right)}{\bar{W}}_{t,\mathrm{tr}}^{\left(V\right)}+\Gamma {\bar{W}}_{t,\mathrm{re}}^{\left(V\right)}\right)}_{y}\left(dx\right), \end{array}$$

where the discrete divergence is as in (47), and Γ=(−1,1) is the matrix consisting of one state change vector corresponding to a forward reaction.

The pair ${\bar{W}}_{t}^{\left(V\right)}:=\left({\bar{W}}_{t,\mathrm{tr}}^{\left(V\right)},{\bar{W}}_{t,\mathrm{re}}^{\left(V\right)}\right)$ is then a Markov process with generator

$$\left(Q^{\left(V\right)}f\right)\left({\bar{w}}_{\mathrm{tr}},{\bar{w}}_{\mathrm{re}}\right):=\left(Q_{\mathrm{tr}}^{\left(V\right)}f\right)\left({\bar{w}}_{\mathrm{tr}},{\bar{w}}_{\mathrm{re}}\right)+\left(Q_{\mathrm{re}}^{\left(V\right)}f\right)\left({\bar{w}}_{\mathrm{tr}},{\bar{w}}_{\mathrm{re}}\right),$$

where

$$\begin{array}{cc} \left(Q_{\mathrm{tr}}^{\left(V\right)}f\right)\left({\bar{w}}_{\mathrm{tr}},{\bar{w}}_{\mathrm{re}}\right) & :=\frac{V}{\epsilon_{V}^{2}}\sum_{y=A,B}D_{y}\;\int \;\phi^{\left(V\right)}{\left[\bar{w}\right]}_{y}\left(dx\right)\sum_{l=1}^{d}(f\left({\bar{w}}_{\mathrm{tr}}-\frac{\epsilon_{V}}{V}\delta_{x-\left(\epsilon_{V}/2\right){𝟙}_{l}}{𝟙}_{y},{\bar{w}}_{\mathrm{re}}\right)\\ & -2f\left({\bar{w}}_{\mathrm{tr}},{\bar{w}}_{\mathrm{re}}\right)+f\left({\bar{w}}_{\mathrm{tr}}+\frac{\epsilon_{V}}{V}\delta_{x+\left(\epsilon_{V}/2\right){𝟙}_{l}}{𝟙}_{y},{\bar{w}}_{\mathrm{re}}\right)),\\ \left(Q_{\mathrm{re}}^{\left(V\right)}f\right)\left({\bar{w}}_{\mathrm{tr}},{\bar{w}}_{\mathrm{re}}\right) & :=V\kappa_{\mathrm{fw}}\int \;\phi^{\left(V\right)}{\left[\bar{w}\right]}_{A}\left(dx\right)\left(f\left({\bar{w}}_{\mathrm{tr}},{\bar{w}}_{\mathrm{re}}+\frac{1}{V}\delta_{x}\right)-f\left({\bar{w}}_{\mathrm{tr}},{\bar{w}}_{\mathrm{re}}\right)\right)\\ & \;+V\kappa_{\mathrm{bw}}\int \;\phi^{\left(V\right)}{\left[\bar{w}\right]}_{B}\left(dx\right)\left(f\left({\bar{w}}_{\mathrm{tr}},{\bar{w}}_{\mathrm{re}}-\frac{1}{V}\delta_{x}\right)-f\left({\bar{w}}_{\mathrm{tr}},{\bar{w}}_{\mathrm{re}}\right)\right). \end{array}$$

### 6.2. Limit and Large Deviations

By the same procedure as in Section 2.3 and Section 5.2, one finds that as V→∞ and $\epsilon_{V}\to 0$, the continuity operator (56) converges to (assuming $\phi {\left[\bar{w}\right]}_{y}\left(dx\right)=\phi {\left[\bar{w}\right]}_{y}\left(x\right)\;dx$):

(57) $$\phi {\left[\bar{w}\right]}_{y}\left(x\right):=\rho_{0,y}\left(x\right)-{\mathrm{div}}_{x}{\bar{w}}_{\mathrm{tr},y}\left(x\right)+{\left(\Gamma {\bar{w}}_{\mathrm{re}}\right)}_{y}\left(x\right),$$

and the process converges (pathwise in probability) to the solution of the system:

$$\begin{array}{cc} {\dot{\bar{w}}}_{t,\mathrm{tr},y}\left(x\right) & =-D_{y}\nabla_{x}\phi {\left[{\bar{w}}_{t}\right]}_{y}\left(x\right),\\ {\dot{\bar{w}}}_{t,\mathrm{re}}\left(x\right) & =\kappa_{\mathrm{fw}}\phi {\left[{\bar{w}}_{t}\right]}_{A}\left(x\right)-\kappa_{\mathrm{bw}}\phi {\left[{\bar{w}}_{t}\right]}_{B}\left(x\right). \end{array}$$

Indeed, putting these together yields the reaction-diffusion equation for the limit concentrations:

$$\begin{array}{c} {\dot{\rho }}_{t,A}\left(x\right)=D_{A}\Delta_{x}\rho_{t,A}\left(x\right)-\kappa_{\mathrm{fw}}\rho_{t,A}\left(x\right)+\kappa_{\mathrm{bw}}\rho_{t,B}\left(x\right),\\ {\dot{\rho }}_{t,B}\left(x\right)=D_{B}\Delta_{x}\rho_{t,B}\left(x\right)+\kappa_{\mathrm{fw}}\rho_{t,A}\left(x\right)-\kappa_{\mathrm{bw}}\rho_{t,B}\left(x\right). \end{array}$$

To find the corresponding large deviations, we combine (17) and (50) to calculate the non-linear generator:

$$\begin{array}{c} \left({ℋ}^{\left(V\right)}f\right)\left({\bar{w}}_{\mathrm{tr}},{\bar{w}}_{\mathrm{re}}\right):=\frac{1}{V}e^{-Vf({\bar{w}}_{\mathrm{tr}},{\bar{w}}_{\mathrm{re}})}\left(Q^{\left(V\right)}e^{Vf}\right)\left({\bar{w}}_{\mathrm{tr}},{\bar{w}}_{\mathrm{re}}\right)\\ \overset{V\to \infty }{\to }\sum_{y=A,B}D_{y}\int \;\left({\mathrm{div}}_{x}\partial_{{\bar{w}}_{\mathrm{tr},y}}f\left({\bar{w}}_{\mathrm{tr}},{\bar{w}}_{\mathrm{re}}\right)\left(x\right)+{\left|\partial_{{\bar{w}}_{\mathrm{tr},y}}f\left({\bar{w}}_{\mathrm{tr}},{\bar{w}}_{\mathrm{re}}\right)\left(x\right)\right|}^{2}\right)\;\phi {\left[\bar{w}\right]}_{y}\left(dx\right)\\ +\kappa_{\mathrm{fw}}\int \;\phi {\left[\bar{w}\right]}_{A}\left(dx\right)\left(e^{\partial_{{\bar{w}}_{\mathrm{re}}}f\left({\bar{w}}_{\mathrm{tr}},{\bar{w}}_{\mathrm{re}}\right)}-1\right)+\kappa_{\mathrm{bw}}\int \;\phi {\left[\bar{w}\right]}_{B}\left(dx\right)\left(e^{-\partial_{{\bar{w}}_{\mathrm{re}}}f\left({\bar{w}}_{\mathrm{tr}},{\bar{w}}_{\mathrm{re}}\right)}-1\right). \end{array}$$

Let us again abbreviate $\bar{\zeta }=\left({\bar{\zeta }}_{\mathrm{tr},y,l}\left(x\right),{\bar{\zeta }}_{\mathrm{re}}\left(x\right)\right)$ and $\bar{j}=\left({\bar{j}}_{\mathrm{tr},y,l}\left(x\right),{\bar{j}}_{\mathrm{re}}\left(x\right)\right)$. The limiting non-linear generator can now be split into

(58) $$\begin{array}{c} ℋ\left(\bar{w},\bar{\zeta }\right):={ℋ}_{\mathrm{tr}}\left(\bar{w},{\bar{\zeta }}_{\mathrm{tr}}\right)+{ℋ}_{\mathrm{re}}\left(\bar{w},{\bar{\zeta }}_{\mathrm{re}}\right),\\ \;{ℋ}_{\mathrm{tr}}\left(\bar{w},{\bar{\zeta }}_{\mathrm{tr}}\right):=\sum_{y=A,B}D_{y}\left({∥{\bar{\zeta }}_{\mathrm{tr},y}∥}_{L^{2}\left(\phi {\left[\bar{w}\right]}_{y}\right)}^{2}-\langle {\bar{\zeta }}_{\mathrm{tr},y},\nabla_{x}\phi {\left[\bar{w}\right]}_{y}\rangle \right),\\ \;{ℋ}_{\mathrm{re}}\left(\bar{w},{\bar{\zeta }}_{\mathrm{re}}\right):=\kappa_{\mathrm{fw}}\int \;\phi {\left[\bar{w}\right]}_{A}\left(dx\right)\left(e^{{\bar{\zeta }}_{\mathrm{re}}\left(x\right)}-1\right)+\kappa_{\mathrm{bw}}\int \;\phi {\left[\bar{w}\right]}_{B}\left(dx\right)\left(e^{-{\bar{\zeta }}_{\mathrm{re}}\left(x\right)}-1\right). \end{array}$$

Since each mechanism corresponds to a separate flux, the corresponding L-function also splits into two parts:

(59) $$\begin{array}{c} ℒ\left(\bar{w},\bar{j}\right):=\underset{{\bar{\zeta }}_{\mathrm{tr}},{\bar{\zeta }}_{\mathrm{re}}}{\sup}\;\langle {\bar{\zeta }}_{\mathrm{tr}},{\bar{j}}_{\mathrm{tr}}\rangle +\langle {\bar{\zeta }}_{\mathrm{re}},{\bar{j}}_{\mathrm{re}}\rangle -ℋ\left(\bar{w},\bar{\zeta }\right):={ℒ}_{\mathrm{tr}}\left(\bar{w},{\bar{j}}_{\mathrm{tr}}\right)+{ℒ}_{\mathrm{re}}\left(\bar{w},{\bar{j}}_{\mathrm{re}}\right),\\ \;{ℒ}_{\mathrm{tr}}\left(\bar{w},{\bar{j}}_{\mathrm{tr}}\right):=\sum_{y=A,B}\frac{1}{4D_{y}}{∥{\bar{j}}_{\mathrm{tr},y}+D_{y}\nabla_{x}\phi {\left[\bar{w}\right]}_{y}∥}_{L^{2}\left(1/\phi {\left[\bar{w}\right]}_{y}\right)}^{2},\\ \;{ℒ}_{\mathrm{re}}\left(\bar{w},{\bar{j}}_{\mathrm{re}}\right):=\underset{j_{\mathrm{fw}}-j_{\mathrm{bw}}={\bar{j}}_{\mathrm{re}}}{\inf}h\left(j_{\mathrm{fw}}|\kappa_{\mathrm{fw}}\phi {\left[\bar{w}\right]}_{A}\right)+h\left(j_{\mathrm{bw}}|\kappa_{\mathrm{bw}}\phi {\left[\bar{w}\right]}_{B}\right), \end{array}$$

using the usual the relative entropy between two measures, i.e.,: h(j|k):=∫j(dx)log(dj/dk(x))−j(dx)+k(dx) if j≪k, else h(j|k):=∞.

As before, the calculation above formally shows that the flux large-deviation principle holds (see for example [32] for a similar but rigorous result):

$${\mathrm{Prob}}^{\left(V\right)}\;\left({\bar{W}}_{\left(\cdot \right)}^{\left(V\right)}\approx {\bar{w}}_{\left(\cdot \right)}\right)\overset{V\to \infty }{∼}e^{-V\int_{0}^{T}\;ℒ\left({\bar{w}}_{t},{\dot{\bar{w}}}_{t}\right)\;dt}.$$

The L-function (59) splits into two parts because the only interaction between the two mechanisms occurs through the state $\phi [\bar{w}]$. By contrast, the corresponding state space large-deviation are much more complicated. Observe that the continuity Equation (57) is an affine function of $\bar{w}$, and so $d\phi_{\bar{w}}\bar{j}$ is independent of $\bar{w}$, and the invariance condition (28) holds. As explained in the beginning of Section 4, this means that one can apply a straightforward contraction principle on the tangents to yield the large deviation cost function for the states/concentrations:

(60) $$\begin{array}{cc} \hat{ℒ}\left(\phi \left[\bar{w}\right],s\right) & :=\underset{\begin{array}{c} \bar{j}=\left({\bar{j}}_{\mathrm{tr}},{\bar{j}}_{\mathrm{re}}\right):\\ s=-\mathrm{div}{\bar{j}}_{\mathrm{tr}}+\Gamma {\bar{j}}_{\mathrm{re}} \end{array}}{\inf}ℒ\left(\bar{w},\bar{j}\right). \end{array}$$

This infimum reintroduces a strong interrelation between the two driving mechanisms. Indeed, for a given tangent (ρ,s), the fluxes in this infimum correspond to an optimal splitting between the two mechanisms, which can be seen as an inf-convolution. Similar interactions also arise when considering multiple reaction pairs, see (Section 3.4, [16]).

### 6.3. GGSs in Flux and State Space

We now apply Theorem 1 to the reaction-diffusion setting. The symmetry condition (34) holds for the function (58) if we choose the free energy functional

$$\begin{array}{c} F\left(\bar{w}\right):=\frac{1}{2}\int \;\phi {\left[\bar{w}\right]}_{A}\left(dx\right)\log\kappa_{\mathrm{fw}}\phi {\left[\bar{w}\right]}_{A}\left(x\right)-\phi {\left[\bar{w}\right]}_{A}\left(dx\right)+\frac{1}{2}\int \;\phi {\left[\bar{w}\right]}_{B}\left(dx\right)\log\kappa_{\mathrm{bw}}\phi {\left[\bar{w}\right]}_{B}\left(x\right)-\phi {\left[\bar{w}\right]}_{B}\left(dx\right). \end{array}$$

Naturally, this functional can be seen as a combination of (22) and (53), where just like (53), it has the form of a relative entropy with respect to a locally finite invariant measure, namely ${\left(\pi_{y}\left(dx\right)\right)}_{y=A,B}\equiv \left(1/\kappa_{\mathrm{fw}},1/\kappa_{\mathrm{bw}}\right)\;dx$. We find the corresponding dissipation potentials from (43), which is again a combination of the non-quadratic potentials (23), (24) and the quadratic potential (54):

(61) $$\begin{array}{c} \Psi^{*}\left(\bar{w},\bar{\zeta }\right):=\Psi_{\mathrm{tr}}^{*}\left(\bar{w},{\bar{\zeta }}_{\mathrm{tr}}\right)+\Psi_{\mathrm{re}}^{*}\left(\bar{w},{\bar{\zeta }}_{\mathrm{re}}\right),\\ \;\Psi_{\mathrm{tr}}^{*}\left(\bar{w},{\bar{\zeta }}_{\mathrm{tr}}\right):=D_{A}{∥{\bar{\zeta }}_{\mathrm{tr},A}∥}_{L^{2}\left(\phi {\left[\bar{w}\right]}_{A}\right)}^{2}+D_{B}{∥{\bar{\zeta }}_{\mathrm{tr},B}∥}_{L^{2}\left(\phi {\left[\bar{w}\right]}_{B}\right)}^{2},\\ \;\Psi_{\mathrm{re}}^{*}\left(\bar{w},{\bar{\zeta }}_{\mathrm{re}}\right):=\int \;\sigma \left(\bar{w}\right)\left(x\right)\left(\cosh({\bar{\zeta }}_{\mathrm{re}}\left(x\right))-1\right)\;dx,\;\;\mathrm{and}\\ \Psi \left(\bar{w},\bar{j}\right):=\Psi_{\mathrm{tr}}\left(\bar{w},{\bar{j}}_{\mathrm{tr}}\right)+\Psi_{\mathrm{re}}\left(\bar{w},{\bar{j}}_{\mathrm{re}}\right),\\ \;\Psi_{\mathrm{tr}}\left(\bar{w},{\bar{j}}_{\mathrm{tr}}\right):=\frac{1}{4D_{A}}{∥{\bar{j}}_{\mathrm{tr},A}∥}_{L^{2}\left(1/\phi {\left[\bar{w}\right]}_{A}\right)}^{2}+\frac{1}{4D_{B}}{∥{\bar{j}}_{\mathrm{tr},B}∥}_{L^{2}\left(1/\phi {\left[\bar{w}\right]}_{B}\right)}^{2},\\ \;\Psi_{\mathrm{re}}\left(\bar{w},{\bar{j}}_{\mathrm{re}}\right):=\int \;\sigma \left(\bar{w}\right)\left(x\right)\left(\cosh^{*}\left({\bar{j}}_{\mathrm{re}}\left(x\right)\sigma \left(\bar{w}\right)\left(x\right)\right)+1\right)\;dx, \end{array}$$

with $\sigma \left(\bar{w}\right)\left(x\right):=2\sqrt{\kappa_{\mathrm{fw}}\kappa_{\mathrm{bw}}\phi {\left[\bar{w}\right]}_{A}\left(x\right)\phi {\left[\bar{w}\right]}_{B}\left(x\right)}$. Let the flux space be given by $W=M_{1}\left(R^{d};R^{d}\right)\times M_{1}\left(R^{d};R^{d}\right)\times L^{1}\left(R^{d}\right)$, where the first two spaces, corresponding to the transport fluxes, are equipped with the metric (55) introduced in the previous section. By Theorem 1 the flux cost function *ℒ* induces the GGS (W,Ψ,F). We stress that the dissipation potential Ψ splits into two potentials for the transport and reaction mechanisms respectively, but the free energy is one and the same for both mechanisms.

Since a GGS is a special case of a pGGEN, by Theorem 3, the state cost function $\hat{ℒ}$ also induces a GGS $\left(X,\hat{\Psi },\hat{F}\right)$, in this case in the space $X=P_{2}\left(R^{d}\times \left\{A,B\right\}\right)$. The same result yields

$$\hat{F}\left(\rho \right):=\frac{1}{2}\int \;\rho_{A}\left(dx\right)\log\kappa_{\mathrm{fw}}\rho_{A}\left(x\right)-\rho_{A}\left(dx\right)+\frac{1}{2}\int \;\rho_{B}\left(dx\right)\log\kappa_{\mathrm{bw}}\rho_{B}\left(x\right)-\rho_{B}\left(dx\right),$$

and, using $d\phi_{\bar{w}}^{T}=\left(\begin{array}{cc} \nabla_{x} & 0\\ 0 & \nabla_{x}\\ -1 & 1 \end{array}\right)$:

(62) $$\begin{array}{cc} {\hat{\Psi }}^{*}\left(\rho ,\xi \right) & \overset{\left(44\right)}{=}\Psi^{*}\left(\bar{w},d\phi_{\bar{w}}^{T}\xi \right)=\Psi_{\mathrm{tr}}^{*}\left(\bar{w},\nabla_{x}\xi \right)+\Psi_{\mathrm{re}}^{*}\left(\bar{w},\xi_{B}-\xi_{A}\right)\\ & \;=D_{A}{∥\nabla_{x}\xi_{A}∥}_{L^{2}\left(\rho_{A}\right)}^{2}+D_{B}{∥\nabla_{x}\xi_{B}∥}_{L^{2}\left(\rho_{B}\right)}^{2}\\ & \;+2\int \;\sqrt{\kappa_{\mathrm{fw}}\kappa_{\mathrm{bw}}\rho_{A}\left(x\right)\rho_{B}\left(x\right)}\left(\cosh(\xi_{B}\left(x\right)-\xi_{A}\left(x\right))-1\right)\;dx,\\ \hat{\Psi }\left(\rho ,s\right)\; & \;\;\;\;\overset{\left(44),(57\right)}{=}\underset{s=-{\mathrm{div}}_{x}{\bar{j}}_{\mathrm{tr}}+\Gamma {\bar{j}}_{\mathrm{re}}}{\inf}\Psi_{\mathrm{tr}}\left(\bar{w},{\bar{j}}_{\mathrm{tr}}\right)+\Psi_{\mathrm{re}}\left(\bar{w},{\bar{j}}_{\mathrm{re}}\right). \end{array}$$

We stress that, analogous to the L-function (60), the dissipation potential $\hat{\Psi }$ on state space no longer splits into two parts.

## 7. Discussion

### 7.1. General Theory

We studied gradient and (pre-) GENERIC structures induced by flux large deviations, and the relationship between structures induced by state large deviations. At a first glance, the physical interpretation of the resulting GGS/GGEN structures in flux space is not immediate. However, in practice many induced flux GGS/GGEN structures have a free energy and dissipation potential that only depends on the integrated flux through the state of the system. Hence the main difference with state space GGSs/GGENs is that the fluxes rather than velocities are being driven, which seems a very physical assumption.

The general theory that we developed in Section 4 presumes two given L-functions, in flux and state space, where the second is related to the first through an infimum; this is the typical setting for large deviation-based cost functions. A condition that is central to this theory is that this infimum can be taken over the second (time-derivative) argument only. When the two L-functions are indeed large-deviation costs, then this condition means that the jump rate in flux space depends on the state only, and not on the integrated flux, which is a very natural assumption as well.

As described in the introduction, one motivation behind this research was to study whether large deviations can induce a GGS/GGEN structure in the flux space when it fails to induce such structure in the state space. This has to be answered negatively. It turns out that if the flux large deviations induce a GGS/GGEN, then so do the state large deviations. The same principle also holds in the other direction, but the flow in flux space could have an additional ‘divergence’-free Hamiltonian term that is not observed when considering states only.

### 7.2. New Structures in Flux Space

Nevertheless, we uncovered a number of previously unknown GGSs/GGENs (as far as the author is aware). The interesting feature of their corresponding dissipation potentials in flux space, is that, due to Equations (44), they have much simpler expresssions than their counterparts in state space.

The first new flux structure that we uncovered is the pGGEN $\left(R_{+}^{R},\Psi ,F,\tilde{k}\circ \phi \right)$ for multiscale chemical reaction networks, where the dissipation potential and free energy is given by (24) and (22). In order for this structure to be a pGGEN, we needed to assume that the fast dynamics do not influence the state, i.e., condition (20). From (20) we see that this condition is sufficient but not necessary, so there is room for possible generalisations.

The second new GGS that we identified is the flux counterpart of the Entropy-Wasserstein gradient flow a described in Section 5.3 and Section 5.4. Much of the (well-developped) Wasserstein calculus is based on transforming to the flux space, performing manipulations and limits there, and then transforming back to the state space, see for example [33,34]. Such arguments may be simplified considerably when working in the flux space directly. It would be interesting to see which geometric properties the new manifold has, e.g., in terms of Ricci curvature bounds and geodesic convexity of the free energy [35].

The third new GGS structure that we derived is a combination of the other two, and models reaction-diffusion equations via their reaction and transport fluxes. Here, we restricted to very simple independent unimolecular reactions, which is certainly generalisable. However, more general reactions would require introducing even more notation as well as more different scaling transitions to circumvent the aforementioned convergence problems. This is beyond the scope of the current paper.

### 7.3. Possible Lines of Future Research

Apart from the open questions mentioned above, the transition from pGGEN to GGEN is worth studying. Throughout the paper we mostly worked with pGGEN rather than GGEN. The reason is that a given L-function can uniquely induce a pGGEN structure, with many different GGEN structures corresponding to it [9,10]. It is still an open question whether and how a meaningful Hamiltonian energy E and Poisson structure *L* in GGEN can be derived from a given drift term *b* in pGGEN such that b(w)=L(w)dE(w) (other than by physical arguments). As found in [10], the Hamiltonian part is often related to dynamics that are deterministic even on the microscopic scale, which also coincides with [7]. However, the setting of multiscale reaction fluxes from Section 3 shows that the Hamiltonian part could come from stochastic dynamics that happen on a faster time scale. Therefore, a possible line of future research would be to consider large deviations on the faster time scale (so-called moderate deviations), and study whether the corresponding variational expression can somehow be used to derived a Hamiltonian structure (W,L,E).

Another line of future research could be to study how structures in flux space can be exploited numerically. As we saw in the examples and Section 6 in particular, if a microscopic system consists of multiple driving mechanisms, then the corresponding Markov generator as well as the non-linear generator is a sum over these mechanisms. By considering separate fluxes for each of these mechanisms, the large-deviation L-function and its induced dissipation potential also splits into different terms for each mechanism. By contrast, when working on the state space this would lead to an inf-convolution (compare for example (61) with (62)). The decomposition that occurs in the space of fluxes can be beneficial for numerical purposes, e.g., using operator splitting techniques [36].

## Abbreviations

The following abbreviations are used in this manuscript:

GENERIC	General Equation for Non-Equilibrium Reversible-Irreversible Coupling
GGS	Generalised Gradient System
GGEN	Generalised GENERIC
pGGEN	pre-(Generalised GENERIC)
MFT	Macroscopic Fluctuation Theory
